# Polygenic background contributes to *GCK*-MODY clinical presentation and glycaemic variability

**DOI:** 10.1007/s00125-026-06712-7

**Published:** 2026-03-18

**Authors:** Jacques Murray Leech, Ankit M. Arni, V. Kartik Chundru, Luke N. Sharp, Kevin Colclough, Andrew T. Hattersley, Michael N. Weedon, Kashyap A. Patel

**Affiliations:** 1 Department of Clinical and Biomedical Sciences, Faculty of Health and Life Sciences, University of Exeter, Exeter, UK; 2 Exeter Genomics Laboratory, Royal Devon University Healthcare NHS Foundation Trust, Exeter, UK

**Keywords:** Diabetes, Genetics, Genetic risk, Glucokinase, HbA_1c_, MODY, Monogenic diabetes, Polygenic risk, Precision medicine

## Abstract

**Aims/hypothesis:**

*GCK*-MODY (glucokinase MODY) causes lifelong, mild hyperglycaemia with high penetrance. Variation in glycaemic phenotype among carriers remains unexplained. We hypothesised that polygenic background contributes to this variability and that this influence differs from that of *HNF1A*-MODY.

**Methods:**

To test whether polygenic background contributes to the *GCK*-MODY clinical phenotype, we analysed polygenic risk scores (PGS) for nine diabetes-related traits in 897 clinically referred individuals with *GCK*-MODY. We compared these to 7645 non-diabetic control participants, 4773 participants with type 2 diabetes and 601 participants with *HNF1A*-MODY and assessed associations between PGS and glycaemic measures. Additionally, we evaluated 158 clinically unselected *GCK* variant carriers from the UK Biobank to examine polygenic effects independent of clinical referral.

**Results:**

We observed independent polygenic enrichment for HbA_1c_ (including both glycaemic and non-glycaemic components), fasting glucose and type 2 diabetes in clinically referred *GCK*-MODY individuals compared with non-diabetes controls (0.16–0.33 SD higher, all *p*<0.003), but not for type 1 diabetes. Importantly, HbA_1c_ and fasting glucose PGSs were higher than in both type 2 diabetes and *HNF1A*-MODY groups, whereas type 2 diabetes PGS was lower. *GCK*-MODY and *HNF1A*-MODY showed distinct patterns of polygenic enrichment, with only the type 2 diabetes PGS contributing independently in *HNF1A*-MODY (0.33 SD, *p*<1 × 10⁻^27^). By contrast, no polygenic enrichment was seen in *GCK* pathogenic variant carriers from a clinically unselected population-based cohort. In both settings, HbA_1c_ PGS were associated with measured HbA_1c_ levels in *GCK* carriers (clinically referred: β=0.97, clinically unselected: β=0.91, both *p*<0.009), with effect sizes similar to those in non-carriers. *GCK*-MODY cases in the top HbA_1c_ quintile had a 3-to-6-fold risk of exceeding the diabetes diagnostic HbA_1c_ threshold (≥48 mmol/mol) in clinically selected and clinically unselected cohort respectively.

**Conclusions/interpretation:**

Our findings suggest that polygenic background and *GCK* variants interact to modify the glycaemic expression of *GCK*-MODY, influencing clinical diagnosis despite high penetrance. The pattern of polygenic contribution differs from that of *HNF1A*-MODY, highlighting the aetiology specific interaction. Our study highlights the importance of integrating both monogenic and polygenic factors to better understand phenotypic variability in monogenic diseases.

**Graphical Abstract:**

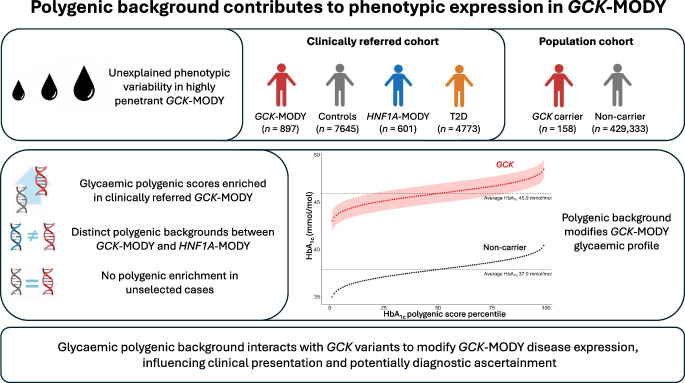

**Supplementary Information:**

The online version contains peer-reviewed but unedited supplementary material available at 10.1007/s00125-026-06712-7.


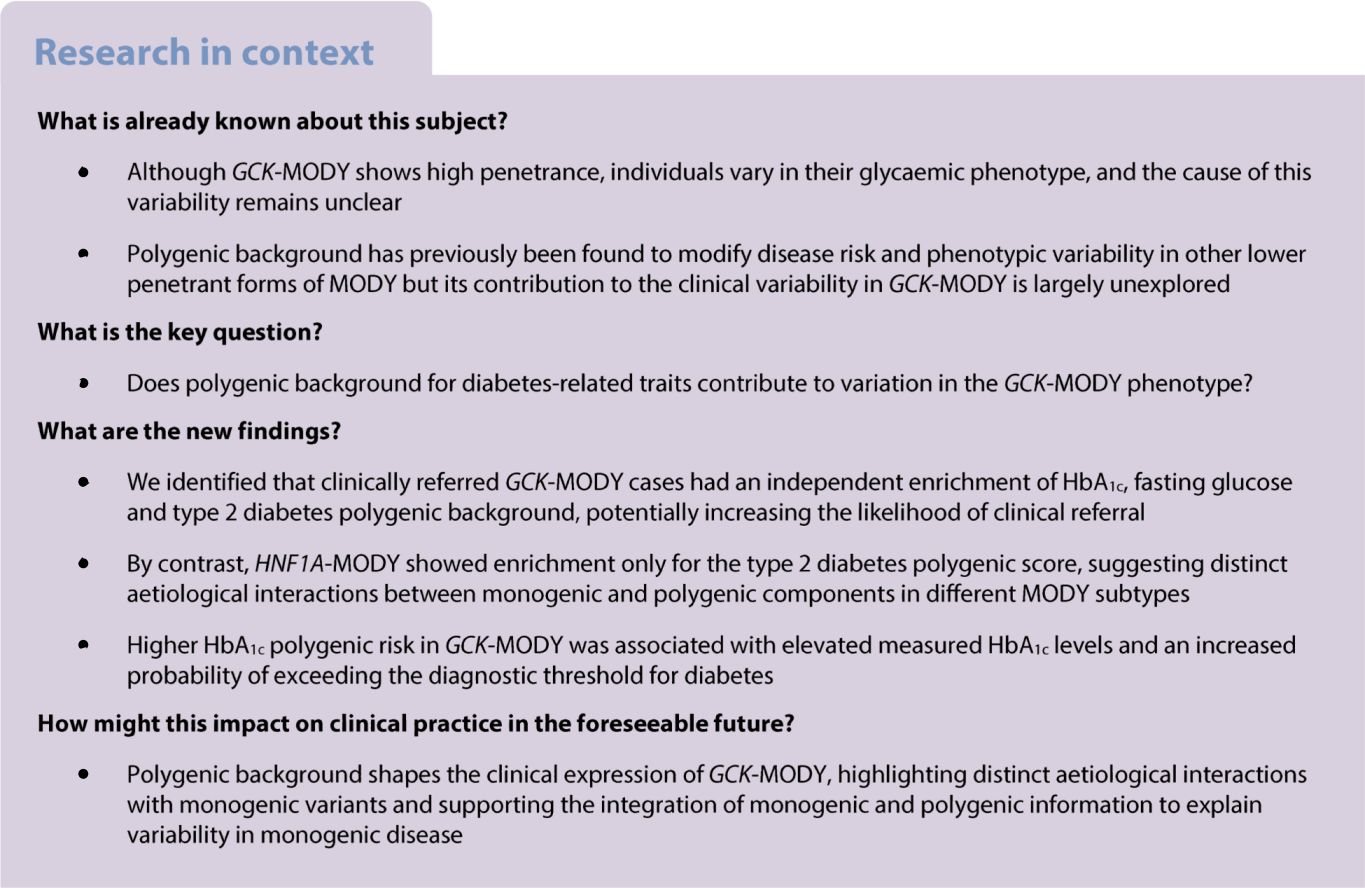


## Introduction

There is growing evidence that polygenic background plays a significant role in shaping the clinical expression of monogenic disorders. These disorders arise from rare, large-effect pathogenic mutations, but population studies have shown that penetrance is often lower than expected in unselected cohorts [1, 2]. Additionally, clinical presentation can vary widely among family members carrying the same causative variant [3, 4], suggesting the presence of modifying genetic or environmental factors [5]. Polygenic background has been found to influence disease risk and presentation in several monogenic disorders, such as kidney disease and long QT syndrome [6, 7]. These findings suggest that rare pathogenic mutations may interact with an individual’s polygenic background to influence the severity, onset and clinical trajectory of monogenic diseases.

Polygenic background has been shown to influence disease expression in monogenic diabetes. MODY is the most common form of monogenic diabetes and is estimated to contribute to 3.6% of all diabetes cases under 30 years of age [8]. Our previous research demonstrated that polygenic background, primarily through type-2-diabetes-associated pathways, can modify the clinical expression of age-dependent MODY subtypes (*HNF1A*, *HNF1B* and *HNF4A)* [9]. In genetically confirmed cases, polygenic burden was strongly associated with earlier diagnosis, greater severity and explained up to 24% of phenotypic variability [9]. Similar findings in smaller cohorts further highlight the substantial influence of polygenic background on these forms of MODY [3, 10]. However, the extent to which polygenic background shapes clinical expression in highly penetrant *GCK*-MODY is unclear.

Variability in the *GCK*-MODY phenotype suggests that polygenic modifiers may influence disease expression. Pathogenic variants in *GCK* are the most common form of monogenic diabetes and account for up to 60% of MODY cases [11]. *GCK*-MODY is marked by lifelong fasting hyperglycaemia, with individuals not demonstrating elevated rates of diabetes-related complications and typically not requiring treatment [11–13]. Diagnosis is usually incidental, following routine HbA_1c_ testing in asymptomatic individuals, frequently leading to misdiagnosis as polygenic forms of diabetes and inappropriate glucose-lowering treatment [11]. *GCK*-MODY has been shown to be highly penetrant, with 96% of carriers having mild hyperglycaemia in the UK Biobank [2]. Despite this, there remains unexplained variability in HbA_1c_ levels in *GCK* carriers (range 38–56 mmol/mol, 5.6–7.3%) [14], which suggests the presence of polygenic modifiers that modify disease expression, even in a highly penetrant monogenic disorder.

Understanding how polygenic background contributes to glycaemic variation in *GCK*-MODY could provide important insights into key biological mechanisms influencing HbA_1c_ levels and glucose regulation. Leveraging the largest clinically referred *GCK*-MODY cohort to date (*N*=897), we used polygenic risk scores (PGS) for type 2 diabetes and related metabolic traits to investigate whether polygenic background contributes to and modifies the clinical presentation of *GCK*-MODY. We replicated our findings in a non-clinically referred cohort of 158 *GCK* carriers from the UK Biobank.

## Research design and methods

### Study populations

#### Clinically referred MODY cohort

We analysed 897 individuals with genetically confirmed *GCK*-MODY referred to the Exeter Genomics Laboratory, Royal Devon University Healthcare NHS Foundation Trust, for monogenic diabetes testing. Most cases originated from the UK and Ireland (642, 71.6%), with 95 additional cases from other European countries (10.6%) and 160 from the rest of the world (17.8%). To compare polygenic risk across differing MODY subtypes, we also included 601 *HNF1A*-MODY probands. Referrals were made through routine clinical care based on suspicion of MODY. Clinical characteristics at referral for genetic testing are detailed in electronic supplementary material (ESM) Table 1. Glycaemic markers were measured by clinicians in their local hospital biochemistry department. All probands or their guardians provided informed consent, and the North Wales Ethics Committee approved the study, with Genetic Beta Cell Research Bank approving sample access (522/WA/0268).

#### Type 2 diabetes cases and non-diabetic controls

Participants with type 2 diabetes (cases) and non-diabetic control participants (controls) were derived from two ethically approved study population-based cohorts, the Exeter 10,000 study [15] and the Diabetes Alliance for Research in England Study [16]. Unselected individuals were recruited via general practices across southwest UK. At recruitment, participants completed baseline health questionnaires and provided fasting blood and urine samples for the measurement of metabolic traits. Type 2 diabetes was defined by clinical diagnosis and lack of insulin treatment or insulin initiation more than 3 years after diagnosis, limiting potential type 1 diabetes inclusion. Non-diabetic controls were defined by no previous diabetes diagnosis and an HbA_1c_ <48 mmol/mol (6.5%) [17]. Analyses were restricted to participants of European ancestry with available genotype data, with participant characteristics provided in ESM Table 1. Access to samples and genotype data was approved by the NIHR Exeter Clinical Research Facility management committee (19/SW/1059).

#### UK Biobank cohort

We used the UK Biobank to assess how polygenic background influences the *GCK*-MODY phenotype when it is not clinically ascertained. The UK Biobank is a large-scale, prospective population-based study with detailed genetic and phenotype data for approximately 500,000 individuals in the UK [18]. Participants were aged between 40 and 70 years at recruitment, and detailed phenotypic data were collected through participant questionnaires, interviews and biomarker measurements [18]. We included 429,491 European individuals who underwent both exome sequencing and array genotyping. Individuals were grouped by the presence of a pathogenic *GCK* variant (*N*=158, ESM Table 2), irrespective of diabetes or glycaemic status. To examine how polygenic risk influenced rates of diabetes-related complications among *GCK* carriers, we defined macrovascular and microvascular complications using a combination of OPCS-4 procedure codes, ICD-10 diagnosis codes, and self-reported data as previously described [19, 20]. All participants provided written informed consent to participate, with ethical approval for the UK Biobank study obtained from the North West Centre for Research Ethics Committee (11/NW/0382).

### Genetic analysis for MODY variants

#### Exeter MODY cohort

All MODY cases were screened for pathogenic variants using either Sanger sequencing or targeted gene panel testing. Assays were performed by the Exeter Genomics Laboratory, Royal Devon University Healthcare NHS Foundation Trust, as part of routine diagnostic testing, with further methodological details outlined previously [21]. Variant interpretation followed the American College of Medical Genetics and Genomics (ACMG)/Association for Molecular Pathology (AMP) guidelines [22], and only individuals with variants classified as likely pathogenic or pathogenic were included. Variants were annotated using the clinically validated transcripts (GenBank: NM_000162.5 for *GCK* and NM_175914.4 for *HNF1A*). Further details on the variant classification protocols used in our local MODY cohort are available in our recent study [2]. A full list of pathogenic *GCK* variants identified in the clinically referred cohort can be found in ESM Table 3.

#### UK Biobank

In the UK Biobank, we used exome sequence data to identify carriers of pathogenic *GCK* variants, following the same transcript and classification guideline as above. In this study, we only included protein-truncating variants (PTVs) deemed to be high confidence by the Loss-Of-Function Transcript Effect Estimator (LOFTEE) [23]. To limit the inclusion of false-positive, low-penetrance variants, we only included missense variants if they were ultra rare in the population (maximum allele count of 2 in gnomAD v2.1.1, minor allele frequency <1.4 × 10–5), had been previously seen in a MODY proband (ClinVar or Exeter Genomics Laboratory) and were classified as pathogenic/likely pathogenic. Sequence read data for all the pathogenic PTV variants were manually reviewed in Integrative Genomics Viewer [24] to exclude false-positive variants. Pathogenic *GCK* variants identified in the UK Biobank cohort are listed in ESM Table 4.

### Array genotyping

#### MODY, type 2 diabetes cases and non-diabetic controls

We used Infinium Global Screening Array genotyping to capture common genetic variation in Exeter MODY and control cohorts. We applied a rigorous quality control pipeline, excluding samples with call rates <98%, sex mismatches (retaining only individuals where reported and genetically inferred sex-matched), relationship discrepancies, or inbreeding coefficients >0.1. Variants with >2% missingness, minor allele frequency <5%, or significant deviation from Hardy–Weinberg equilibrium (*p*<1 × 10⁻^6^) were removed, both within and across batches. We performed genotype imputation using the TOPMed reference panel (Version 2) [25] via the Michigan Imputation Server [26], using LD-pruned variants as input. Genetic ancestry was determined by principal component analysis (PCA), comparing study participants to reference populations from the 1000 Genomes Project (Phase 3) and the Human Genome Diversity Project [27], implemented through the GenoPred pipeline (v2.2.1) [28], with only individuals of European ancestry analysed. Relatedness was assessed using the KING robust algorithm (v2.2.4) [29]. To better model population structure within the cohort, we performed PCA again using FlashPCA (v2.0) [30]. Principal components were first derived from unrelated European individuals and then projected onto related individuals.

#### UK Biobank

UK Biobank individuals were SNP-genotyped using the UK BiLEVE array (~50,000 individuals), and the UKB Axiom Array (~450.000 individuals). This dataset underwent extensive central quality control and was imputed to the TOPMed reference panel [25]. In addition, approximately 450,000 individuals underwent exome sequencing using the IDT xGen Exome Research Panel v1.0. The sequencing and quality control pipeline has been described in detail elsewhere [31]. Briefly, variants were called using GATK v3.0, with filters excluding variants with an inbreeding coefficient <–0.03 or lacking at least one genotype meeting the following thresholds: depth (DP) ≥10, genotype quality (GQ) ≥20, and allele balance (AB) ≥0.20 for heterozygotes. Ancestry was inferred by PCA using the approach applied to our local cohorts.

### Polygenic score calculation

To calculate PGS for nine diabetes-related traits, we followed the methodology described in our previous study [9]. In brief, polygenic scores based on genome-wide significant variants were calculated using the PLINK 1.9’s score function [32]. Where comprehensive summary statistics were available, genome-wide scores were constructed using the GenoPred pipeline (v2.2.1) [28] using the LDpred2 auto model [33], allowing additional genetic signal to be captured. Weights for fasting glucose and HbA_1c_ genetic risk scores were obtained from Chen et al [34]*,* using LD-pruned variants (*r*^2^<0.1) from the Trans-Ancestry and European single-ancestry analyses. To better understand how polygenic background modifies the *GCK* phenotype, these scores were partitioned using overlapping variants from previous type 2 diabetes association studies (Chen et al [34], Table S4) and classified as type 2 diabetes increasing if they had been previously found to have a *p* value <0.05 for type 2 diabetes risk. Similarly, glycaemic and non-glycaemic HbA_1c_ variants were identified using signal classification (Chen et al [34], Table S20). Further details, including the number of variants incorporated and the source studies [34–41], are provided in ESM Table 5. To ensure comparability across datasets and avoid distortion due to case enrichment, each PGS was standardised using the mean and SD of non-diabetic control individuals in each cohort.

### Statistical analysis

To determine if *GCK*-MODY cases carry excess polygenic risk, we compared nine diabetes-related PGSs against controls using linear models adjusted for principal components. Similar comparisons were performed for *HNF1A*-MODY and type 2 diabetes. We initially modelled scores separately, then used multivariable logistic regression to identify independently enriched pathways. As a supplementary analysis, we compared polygenic scores in *GCK*-MODY cases to age- (±1 year) and sex-matched (exact) controls, using Mahalanobis nearest-neighbour matching. Additionally, we compared *GCK*-MODY cases to a sample population with a target type 2 diabetes prevalence of 7% [42]. We generated 500 bootstrap samples of 5000 individuals each, resampling non-diabetic controls and type 2 diabetes cases to achieve the target prevalence, and compared polygenic scores between each resampled population and *GCK*-MODY cases.

To evaluate how polygenic risk influenced the *GCK*-MODY phenotype, we used mixed-effects models to test the association between polygenic scores and key glycaemic outcomes (fasting glucose and HbA_1c_). Family ID was included as a random effect to account for within-family correlations among *GCK*-MODY cases. For control individuals, standard linear models were used as they were unrelated. Each PGS was first assessed in a separate model, adjusting for principal components to account for population structure. Significant PGS were further analysed, adjusting for relevant clinical and genetic covariates that could influence glycaemic outcomes, including sex, age, BMI, parental history of diabetes and mutation type.

Clinically unselected *GCK* variant carriers in the UK Biobank enabled us to assess the impact of polygenic background on glycaemic outcomes in a second, population-based setting. We tested associations between each PGS and HbA_1c_ levels, adjusting for available covariates (sex, age, BMI, parental diabetes, mutation type and genetic principal components). As only the HbA_1c_ PGS was significant, we examined predicted HbA_1c_ across PGS percentiles. We also tested the HbA_1c_ PGS against the likelihood of exceeding the diagnostic HbA_1c_ threshold for diabetes (≥48 mmol/mol, 6.5%) [17]. These associations were examined by comparing individuals in the top, middle and bottom HbA_1c_ PGS quintiles, using both unadjusted and adjusted logistic regression models. Adjusted logistic regression was also used to assess association of polygenic scores and diabetes-related complications.

All statistical analyses were performed using R version 4.4.1.

## Results

### Clinically referred *GCK*-MODY have elevated polygenic risk for glycaemic traits

How factors beyond the primary pathogenic variant influence disease expression in highly penetrant *GCK*-MODY is not clearly understood. We hypothesise that some of the variation in expression even in this highly penetrant disease could be due to polygenic background. To investigate the contribution of polygenic background to *GCK*-MODY we analysed polygenic scores for nine diabetes-related traits in 897 clinically referred patients with *GCK*-MODY (ESM Table 1). As expected, *GCK*-MODY had higher measured HbA_1c_ and fasting glucose compared with 7645 non-diabetic control participants (mean 8.3 mmol/mol and 1.79 mmol/l difference respectively, *p*<1 × 10^−100^).

**Fig. 1:**
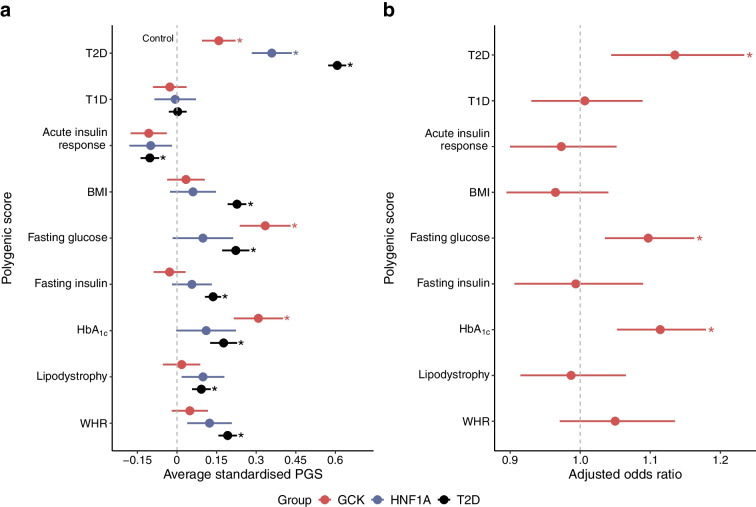
Elevated polygenic risk in clinically referred *GCK*-MODY. (**a**) Standardised differences in nine diabetes-related polygenic scores, each assessed separately using linear regression, adjusting for the first ten genetic ancestry principal components. *GCK*-MODY cases (red, *N*=897), *HNF1A*-MODY cases (blue, *N*=601) and type 2 diabetes cases (black, *N*=4773) are compared against control individuals without diabetes (dashed grey line, *N*=7645). (**b**) Adjusted odds ratios for *GCK*-MODY vs controls, estimated from a multivariable logistic regression model that includes all nine PGS simultaneously, along with the first ten genetic ancestry principal components. All scores are standardised to have a mean of 0 and SD of 1 in controls. Odds ratios represent the change in risk associated with a 1 SD increase in the respective polygenic score. Asterisks denote Bonferroni-adjusted statistically significant differences from controls (**a**: *p*<0.0019, **b**: *p*<0.0056). Dots represent the estimates, with error bars indicating 95% CIs. T1D, type 1 diabetes; T2D, type 2 diabetes; WHR, waist hip ratio

Compared with control participants, *GCK*-MODY patients had significantly higher PGS for type 2 diabetes, HbA_1c_ and fasting glucose (0.16–0.33 SD increase, all *p*<1 × 10^−6^; Fig. 1a). Notably, there was no enrichment for type 1 diabetes PGS (*p*=0.36). To determine whether this enrichment was specific to *GCK*-MODY, we compared these scores with 4773 type 2 diabetes cases and 601 *HNF1A*-MODY probands. The pattern of polygenic contribution varied substantially across disease. Type 2 diabetes cases showed excess risk across multiple pathways, with the strongest effects observed in type 2 diabetes-related pathways (0.61 SD increase, *p*<8.7 × 10^−257^), with lower contributions from HbA_1c_ and fasting glucose pathways than observed in *GCK*-MODY. *HNF1A*-MODY cases had a 0.27 SD higher type 2 diabetes PGS than controls (*p*<1.3 × 10^−27^) but did not show enrichment in glycaemic pathways as observed in *GCK*-MODY, suggesting that the specific pathways influencing clinical presentation are dependent on the underlying monogenic biology.

Sensitivity analysis limiting to probands only (*N*=705) showed consistent findings (ESM Fig. 1). Additional analysis using age- and sex-matched case–controls (*N*=722), or a reference population including 7% type 2 diabetes cases, also showed similar enrichment of polygenic risk in *GCK*-MODY (ESM Figs 2, 3). The type 2 diabetes, HbA_1c_ and fasting glucose PGS were independently enriched after adjusting for other polygenic scores (Fig. 1b), indicating that clinical phenotype in clinically referred cases of *GCK*-MODY may be shaped by both *GCK* variant and glucose-rising biological pathways. To explore this further we stratified the HbA_1c_ and fasting glucose scores by their type 2 diabetes associations and found that both type 2 diabetes and non-type 2 diabetes pathways were enriched in *GCK*-MODY (ESM Fig. 4). Similarly, using a partitioned HbA_1c_ risk score, both glycaemic and non-glycaemic pathways were enriched in *GCK*-MODY (ESM Fig. 4). These data together suggest that diverse polygenic mechanisms that increase HbA_1c_ contribute to the phenotype of the clinically identified *GCK*-MODY.

### Increased polygenic risk modifies HbA_1c_ levels in clinically referred *GCK*-MODY

**Fig. 2:**
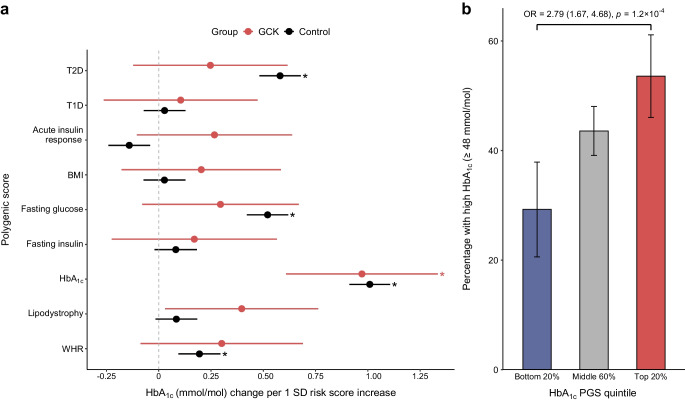
Increased polygenic burden associated with higher HbA_1c_ levels in *GCK*-MODY. (**a**) Association between polygenic scores for nine diabetes-related traits and HbA_1c_ levels (mmol/mol). All scores were assessed individually. For *GCK*-MODY (red, *N*=897), estimates were derived using a mixed-effects linear model with family as a random effect and adjusted for the first ten genetic ancestry principal components. For controls (black, *N*=7645), standard linear regression was used. Estimates represent the effect of a 1 SD increase in the respective polygenic score. Dots represent the estimates, with error bars indicating 95% CIs. Asterisks highlight significant differences after Bonferroni correction (*p*<0.0028). (**b**) Bar plot showing the percentage of *GCK-*MODY cases exceeding the diagnostic threshold for diabetes (HbA_1c_ ≥48 mmol/mol), stratified by HbA_1c_ polygenic score quintiles (bottom 20%, middle 60% and top 20%). In these groups, 31/109, 207/475 and 90/169 cases, respectively, met the diagnostic threshold. Error bars represent 95% CIs. T1D, type 1 diabetes; T2D, type 2 diabetes; WHR, waist hip ratio. Odds ratios derived from unadjusted logistic models comparing the top and bottom HbA_1c_ PGS quintile

Having observed elevated polygenic burden in clinically referred *GCK*-MODY cases, we next assessed whether this burden influences key features of the *GCK*-MODY phenotype (HbA_1c_ or fasting glucose). Among the polygenic scores tested, only the HbA_1c_ PGS showed a significant association with HbA_1c_ levels in *GCK*-MODY (*p*<1.8 × 10^−7^), with a one SD increase in the HbA_1c_ PGS associated with a 0.97 mmol/mol (95% CI 0.61, 1.33 mmol/mol) increase in HbA_1c_ levels (Fig. 2a). The effect size was similar to that in controls (*p* interaction=0.74). This association remained after adjusting for clinical and genotypic information (β=0.78, *p*=4.9 × 10^−7^, ESM Table 6). In addition to the effect of HbA_1c_ PGS, we also noted the independent effects of sex (female individuals 1.8 mmol/mol lower HbA_1c_) and BMI (0.1 mmol/mol increase per kg/m^2^; ESM Table 6). Carriers of *GCK* PTVs had significantly higher HbA_1c_ compared with those with missense variants (mean difference=1.41 mmol/mol, *p*=2.9 × 10^−4^; ESM Fig. 5). When partitioned into glycaemic and non-glycaemic components, the HbA_1c_ association in *GCK*-MODY appeared to be more strongly influenced by the non-glycaemic pathway (β=0.76, *p*=4.3 × 10^−5^), with a similar pattern seen in controls (ESM Fig. 6). We next examined whether polygenic background was associated with the likelihood of meeting the diagnostic threshold for diabetes (HbA_1c_ ≥48 mmol/mol), potentially shaping clinical recognition. In the top HbA_1c_ PGS quintile, 53.3% of *GCK*-MODY cases exceeded the diagnostic threshold, compared with 29% in the lowest quintile (OR=2.79, 95% CI 1.67, 4.68, *p*=1.2 × 10^−4^; Fig. 2b). These associations remained significant after adjusting for clinical and genetic covariates (ESM Table 7). We did not see any significant association between any PGS and fasting blood glucose levels in *GCK*-MODY (ESM Fig. 7).

### Polygenic background modifies HbA_1c_ levels in clinically unselected *GCK* carriers

**Fig. 3:**
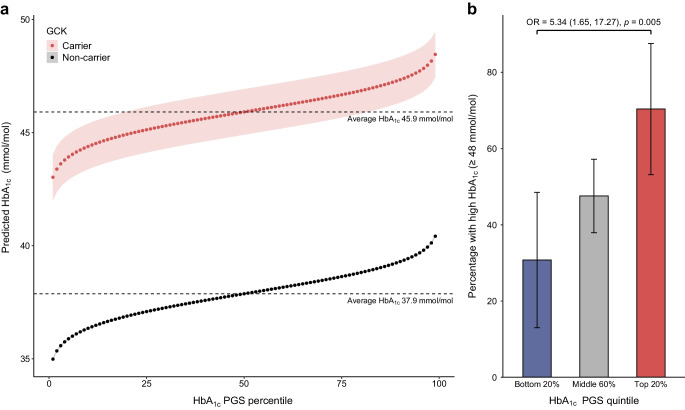
HbA_1c_ PGS modifies phenotype in clinically unselected *GCK* carriers. (**a**) Predicted HbA_1c_ (mmol/mol), assessed using a linear model with HbA_1c_ as a continuous variable adjusted for sex, age, parental history of diabetes, BMI, *GCK* carrier status and mutation type, computing the marginalised effect per percentile. Dots represent percentiles and shaded regions represent 95% CIs. Dashed lines highlight the baseline HbA_1c_ at the 50th percentile for *GCK* carriers (red, *N*=158) and non-carriers (black, *N*=429,333). (**b**) Bar plot showing the percentage of *GCK* cases exceeding the diagnostic threshold for diabetes (HbA_1c_ ≥48 mmol/mol), stratified by HbA_1c_ polygenic score quintiles (bottom 20%, middle 60% and top 20%). In these groups, 8/26, 49/103 and 19/27 carriers, respectively, exceeded the diagnostic threshold. Error bars represent 95% CIs. Odds ratios derived from unadjusted logistic models comparing the top and bottom HbA_1c_ PGS quintile

We observed that clinically referred *GCK*-MODY cases have elevated polygenic burden associated with higher HbA_1c_ levels, which may contribute to clinical recognition. We therefore hypothesised that this enrichment would not appear in unselected *GCK* carriers, although polygenic risk would still influence HbA_1c_ variability. To assess this, we examined individuals carrying pathogenic *GCK* variants (*N*=158) from the UK Biobank population-based cohort (*N*=429,333; ESM Table 2). Compared with non-carriers, unselected *GCK* carriers showed no enrichment in any of polygenic scores previously found to be independently elevated in the clinically referred cohort (all *p*>0.05; ESM Fig. 8). However, as in referred cases, only the HbA_1c_ PGS was significantly associated with HbA_1c_ levels in unselected *GCK* carriers after adjusting for clinical and genetic covariates (ESM Fig. 9). A one SD increase in HbA_1c_ PGS was associated with a 0.91 mmol/mol increase in HbA_1c_ (0.23–1.59 mmol/mol, *p*=0.009). A sensitivity analysis in 141 unrelated *GCK* carriers showed consistent results (ESM Fig. 10). The effect was similar to that in non-carriers (*p* interaction = 0.47). Predicted HbA_1c_ was calculated from the HbA_1c_ PGS, providing an estimate of the polygenic effect on HbA_1c_, independent of clinical and genetic confounders. Predicted HbA_1c_ ranged from 43.08 mmol/mol at the 1st percentile (95% CI 42.02, 44.03 mmol/mol) to 48.46 mmol/mol at the 99th percentile (95% CI 47.45, 49.47 mmol/mol) in *GCK* carriers (Fig. 3a). Individuals without *GCK* variants but with the highest polygenic risk did not reach these HbA_1c_ levels (predicted HbA_1c_=40.42 mmol/mol, 95% CI 40.37, 40.46 mmol/mol), suggesting that extreme polygenic risk alone cannot fully replicate the glycaemic effect of a pathogenic *GCK* variant as seen in other monogenic disorders. Notably, *GCK* carriers in the highest HbA_1c_ PGS quintile had significantly greater odds of exceeding the diabetes diagnostic threshold (HbA_1c_ ≥48 mmol/mol) compared with those in the lowest quintile (OR=5.34, 95% CI 1.65, 17.27, *p*=0.005; Fig. 3b). However, polygenic scores were not statistically associated with microvascular or macrovascular complications in *GCK* carriers (all *p*>0.05, ESM Fig. 11). Comparatively, 30.7% of *GCK* carriers (95% CI 9.1, 48.5%) in the lowest quintile exceed this threshold, compared with just 4.5% (95% CI 4.3, 4.6%) of non-carriers in the highest quintile (*p*=1.32 × 10^−5^). These associations remained robust after adjusting for clinical and genetic covariates (ESM Table 7), further highlighting how polygenic background and monogenic variants interact to shape disease, even in a highly penetrant disorder.

## Discussion

Our analysis reveals that a polygenic background plays a role in shaping the phenotypic expression of highly penetrant *GCK*-MODY. Using data from 1059 carriers of pathogenic *GCK* variants, we show that polygenic risk contributes to glycaemic variability, in both clinically referred and population-based *GCK* carriers.

Despite *GCK*-MODY being a highly penetrant monogenic disorder characterised by stable lifelong hyperglycaemia [11], we observe that polygenic background can influence its clinical expression. Interestingly, our data suggest that the polygenic modifiers contributing to *GCK*-MODY differ from those observed in *HNF1A*-MODY. Our results highlight that *HNF1A*-MODY probands were predominantly influenced by type 2 diabetes polygenic risk (0.27 SD increase). By contrast, clinically referred *GCK*-MODY patients were independently enriched for type 2 diabetes, HbA_1c_ and fasting glucose polygenic risk (all *p*<0.003). These differences overlap with known biological differences between the MODY subtypes, with *HNF1A*-MODY characterised by progressive beta cell decline and *GCK*-MODY by impaired glucose sensing. This pattern suggests that polygenic background has the greatest impact when it converges on biological mechanisms relevant to the underlying monogenic disorder. Among individuals with *GCK*-MODY, partitioned HbA_1c_ PGS indicate that both glycaemic and non-glycaemic pathways contribute to variation in HbA_1c_, influencing both underlying glucose levels and measured HbA_1c_, with implications for clinical ascertainment. We observed a lack of enrichment for type 1 diabetes polygenic risk, consistent with the autoimmune nature of type 1 diabetes and supporting the potential use of type 1 diabetes risk scores to distinguish *GCK*-MODY from type 1 diabetes in clinical settings [43, 44].

This complex interplay between large-effect pathogenic mutation and polygenic modifiers supports a liability threshold model of disease. This probability for crossing the threshold for disease expression is primarily driven by the causative pathogenic variant with risk modified by environmental and polygenic factors [45], now showcased in several monogenic disorders [46]. Since *GCK*-MODY is present from birth and typically identified later in life from incidental glucose testing [11], polygenic background may influence whether an individual is identified and referred for genetic testing. This is supported by our finding that clinically referred *GCK*-MODY patients demonstrated excess polygenic risk, while unselected *GCK* carriers did not, which suggests that polygenic background is a contributing factor to shaping the phenotype that leads to clinical identification and referral. Several studies have demonstrated that increased polygenic risk is associated with increased phenotypic severity, such as in familial epilepsy [47] and long QT syndrome [6]. While diabetes-related complications are typically considered rare in *GCK*-MODY patients [13], polygenic risk could potentially contribute to more severe phenotypes in some individuals. For example, a recent report by Ji et al [48] described a *GCK* proband with severe diabetes features, potentially explained by the high insulin resistance polygenic risk enriched on the maternal side, highlighting the need for dedicated studies to explore this systematically. Additionally, *GCK* individuals are often misdiagnosed as polygenic forms of diabetes, leading to unnecessary treatment. This is supported by our findings that *GCK* carriers with higher polygenic risk are more likely to exceed the diagnostic HbA_1c_ threshold (HbA_1c_ ≥48 mmol/mol). This suggests that misdiagnosis risk may be heightened in individuals with higher polygenic burden, who are more likely to present with a typical diabetes phenotype. Further studies are needed to assess the role of polygenic modifiers in the progression and identification of *GCK*-MODY.

Despite this modifying influence, polygenic background alone was insufficient to replicate the monogenic phenotype of *GCK*-MODY in the general population. Clinically unselected individuals of high HbA_1c_ polygenic risk (99th percentile, HbA_1c_ 40.42 mmol/mol) did not display the glycaemic profile seen in *GCK* carriers of low polygenic risk (1st percentile, HbA_1c_ 43.08 mmol/mol). This differs from conditions like *HNF1A/HNF1B/HNF4A*-MODY [9] and familial hypercholesterolaemia [49], in which high polygenic risk can mimic monogenic phenotypes. The absence of a phenocopy in *GCK*-MODY could be explained by the particularly high penetrance and strong effect size of the causative mutations. By contrast, recent work by Huerta-Chagoya et al [38] demonstrated that non-carriers with high polygenic risk had a similar diabetes risk to carriers of the intermediate effect *GCK* variant p.Val455Glu with low polygenic risk. This suggests that large impact pathogenic mutations in *GCK*-MODY effectively raise the threshold for disease expression, making it difficult for polygenic risk alone to replicate.

This study represents the largest *GCK*-MODY cohort analysed to date, but several limitations should be acknowledged. Our findings are based on UK-based, European ancestry cohorts, limiting generalisability to other populations. Our clinically selected cohort is derived from routine clinical referrals; however, the ascertainment of cases may be influenced by several environmental factors, including healthcare access, socioeconomic status, variability in clinical practices, and other unmeasured confounders. We adjusted our analyses of HbA_1c_ levels for several known or measurable confounding factors, including mutation type, family ID, BMI, age and parental diabetes status. While we have adjusted for known covariates, unmeasured confounders could still potentially affect the associations between PGS and *GCK*-MODY phenotype. Notably, we did not observe an association between polygenic background and fasting glucose levels, which may in part reflect variability in the timing and methodology of glucose measurements across different clinical sites, and lower variability explained by the fasting glucose PGS [34, 50]. While these limitations remain, the large sample size in the clinically selected cohort may mitigate some of their effects. Sample size limitations in the unselected *GCK*-MODY cohort restricted our ability to assess associations with diabetes-related complications and perform detailed subgroup analyses.

In conclusion, we demonstrate that polygenic background modifies the clinical presentation of highly penetrant *GCK*-MODY, with HbA_1c_-related pathways playing a key role in phenotypic variability. Future work in larger and more diverse cohorts is needed to identify specific modifier variants underlying this polygenic influence. Our findings support integrated approaches combining monogenic and polygenic risk information to enhance biological understanding and improve clinical management of monogenic disease.

## Supplementary Information

Below is the link to the electronic supplementary material.

ESM1 (PDF 659 KB)

## Data Availability

The data supporting the findings of this study are available within the article and its supplemental information. The clinical data generated and/or analysed as part of this study are not publicly available because of patient confidentiality and ethical approval associated with the data but are available from the corresponding authors upon reasonable request. The UK Biobank dataset is available from: https://biobank.ctsu.ox.ac.uk

## Abbreviations

*GCK*-MODY: Glucokinase MODY
PCA: Principal component analysis
PGS: Polygenic risk scores
PTVs: Protein-truncating variants
