# Supplementary material for: Polygenic background contributes to GCK-MODY clinical presentation and glycaemic variability
Source: Diabetologia. 2026 Mar 18;69(7):1951–61. doi: 10.1007/s00125-026-06712-7 (PMC13236825; doi:10.1007/s00125-026-06712-7)
Supplement: Supplementary file 1 — ESM1 (PDF 659 KB) [file 125_2026_6712_MOESM1_ESM.pdf]

Electronic supplementary material

Characteristics	Control	GCK-MODY	HNF1A-MODY	T2D
N	7,645	897	601	4773
Female Sex, n (%)	4,849 (63.4%)	577 (64.3%)	408 (67.9%)	1957 (41%)
Age at Recruitment, y	54.3 (15.15)	32.3 (16.5)	39.9 (16.4)	68.3 (10.3)
BMI (kg/m²)	26.48 (4.68)	23.5 (4.4)	24.8 (4.3)	30.8 (5.7)
Parent Diabetes, n (%)				
None	6,205 (82.9%)	184 (22.1%)	72 (12.3%)	1203 (67%)
Mother	571 (7.6%)	347 (41.8%)	268 (45.7%)	227 (15.4%)
Father	621 (8.3%)	269 (32.4%)	200 (34.1%)	243 (13.5%)
Both	84 (1.1%)	31 (3.7%)	32 (3.8%)	73 (4.1%)
HbA_{1c} (mmol/mol)	37.8 (4.4)	46.1 (5.1)	58.9 (17.6)	55.0 (12.2)
Fasting Blood Glucose (mmol/ L)	4.99 (0.57)	6.79 (3.07)	-	-

ESM Table 1: Characteristics of local cohort, at referral for genetic testing (MODY) or recruitment (control/ T2D). For continuous variables, values are presented as mean (SD), and for categorical variables, counts (n) and percentages (%) are provided. BMI = Body Mass Index, y = years.

Characteristics	***GCK***
N	158
Female Sex, n (%)	84 (53.2%)
Age at Recruitment, y	56.6 (8.2)
BMI (kg/m²)	27.9 (4.9)
Parent Diabetes, n (%)	
None	101 (63.9%)
Mother	31 (19.6%)
Father	20 (12.7%)
Both	6 (3.8%)
HbA_{1c} (mmol/mol)	46.9 (4.9)
Fasting Blood Glucose (mmol/L)	6.4 (0.8)
Diabetic Complication, n (%)	
Any	41 (25.9%)
Any Macrovascular	24 (15.2%)
Stroke	8 (5.1%)
Coronary Artery Disease	17 (10.8%)
Any Microvascular	5 (15.8%)
Neuropathy	<5 (<3.2%)
Retinopathy	21 (13.3%)
Kidney Disease	<5 (<3.2%)

ESM Table 2: Clinical Characteristics of UK Biobank *GCK* carriers. Clinical characteristics of *GCK* carriers identified in the UK Biobank participants. For continuous variables, values are presented as mean (SD), and for categorical variables, counts (n) and percentages (%) are provided. BMI = Body Mass Index, y = years. Cell counts fewer than five are reported as <5 to comply with UK Biobank anonymisation guidelines.

Gene	Transcript	DNA nomenclature	Protein nomenclature	Classification
GCK	NM_000162.5	c.(?_-440)_(*332_?)del	p.?	Pathogenic
GCK	NM_000162.5	c.(?_-441)_(45+13_46-1)del	p.?	Pathogenic
GCK	NM_000162.5	c.1-?_45+?del	p.0?	Pathogenic
GCK	NM_000162.5	c.1000_1018del	p.F334fs	Pathogenic
GCK	NM_000162.5	c.1007C>A	p.S336*	Pathogenic
GCK	NM_000162.5	c.1010A>C	p.Q337P	Likely pathogenic
GCK	NM_000162.5	c.1013_1019+47del	p.V338fs	Pathogenic
GCK	NM_000162.5	c.1018A>G	p.S340G	Likely pathogenic
GCK	NM_000162.5	c.1019+1G>A	p.?	Pathogenic
GCK	NM_000162.5	c.1019G>A	p.S340N	Likely pathogenic
GCK	NM_000162.5	c.106C>T	p.R36W	Likely pathogenic
GCK	NM_000162.5	c.1079C>A	p.S360*	Likely pathogenic
GCK	NM_000162.5	c.107G>C	p.R36P	Likely pathogenic
GCK	NM_000162.5	c.1092C>A	p.C364*	Pathogenic
GCK	NM_000162.5	c.1092C>G	p.C364W	Likely pathogenic
GCK	NM_000162.5	c.1099G>A	p.V367M	Pathogenic
GCK	NM_000162.5	c.110T>G	p.M37R	Pathogenic
GCK	NM_000162.5	c.1113C>G	p.C371W	Likely pathogenic
GCK	NM_000162.5	c.1115_1137dup	p.H380fs	Pathogenic
GCK	NM_000162.5	c.1119_1120dup	p.V374fs	Pathogenic
GCK	NM_000162.5	c.1124C>T	p.S375F	Likely pathogenic
GCK	NM_000162.5	c.1130G>T	p.R377L	Likely pathogenic
GCK	NM_000162.5	c.1132G>A	p.A378T	Pathogenic
GCK	NM_000162.5	c.1132G>C	p.A378P	Likely pathogenic
GCK	NM_000162.5	c.1133C>A	p.A378D	Pathogenic
GCK	NM_000162.5	c.1133C>T	p.A378V	Pathogenic
GCK	NM_000162.5	c.1136C>T	p.A379V	Likely pathogenic
GCK	NM_000162.5	c.1142T>A	p.M381K	Likely pathogenic
GCK	NM_000162.5	c.1142T>C	p.M381T	Pathogenic
GCK	NM_000162.5	c.1142T>G	p.M381R	Likely pathogenic
GCK	NM_000162.5	c.1144T>C	p.C382R	Pathogenic
GCK	NM_000162.5	c.1145G>A	p.C382Y	Pathogenic
GCK	NM_000162.5	c.1148C>T	p.S383L	Pathogenic
GCK	NM_000162.5	c.115_117del	p.K39del	Likely pathogenic
GCK	NM_000162.5	c.1150G>A	p.A384T	Likely pathogenic
GCK	NM_000162.5	c.1153G>A	p.G385R	Likely pathogenic
GCK	NM_000162.5	c.1159G>A	p.A387T	Pathogenic
GCK	NM_000162.5	c.1160C>A	p.A387E	Likely pathogenic
GCK	NM_000162.5	c.1160C>T	p.A387V	Pathogenic
GCK	NM_000162.5	c.1166T>A	p.V389D	Likely pathogenic
GCK	NM_000162.5	c.1169_1171del	p.I390del	Likely pathogenic
GCK	NM_000162.5	c.1174C>T	p.R392C	Pathogenic
GCK	NM_000162.5	c.1175G>C	p.R392P	Likely pathogenic
GCK	NM_000162.5	c.1183G>T	p.E395*	Pathogenic

GCK	NM_000162.5	c.118G>A	p.E40K	Pathogenic
GCK	NM_000162.5	c.1190G>T	p.R397L	Pathogenic
GCK	NM_000162.5	c.1209del	p.I404fs	Pathogenic
GCK	NM_000162.5	c.121A>G	p.M41V/N	Likely pathogenic
GCK	NM_000162.5	c.1221_1225del	p.V408fs	Pathogenic
GCK	NM_000162.5	c.1226del	p.D409fs	Pathogenic
GCK	NM_000162.5	c.1228G>C	p.G410R	Pathogenic
GCK	NM_000162.5	c.1228G>T	p.G410C	Pathogenic
GCK	NM_000162.5	c.1229G>A	p.G410D	Pathogenic
GCK	NM_000162.5	c.122T>C	p.M41T	Likely pathogenic
GCK	NM_000162.5	c.1232_1244del	p.S411fs	Pathogenic
GCK	NM_000162.5	c.1234G>A	p.V412M	Pathogenic
GCK	NM_000162.5	c.1235T>A	p.V412E	Pathogenic
GCK	NM_000162.5	c.1260G>T	p.K420N	Likely pathogenic
GCK	NM_000162.5	c.1261G>T	p.E421*	Pathogenic
GCK	NM_000162.5	c.1264C>T	p.R422W	Likely pathogenic
GCK	NM_000162.5	c.1270C>T	p.H424Y	Likely pathogenic
GCK	NM_000162.5	c.127C>T	p.R43C	Pathogenic
GCK	NM_000162.5	c.128G>A	p.R43H	Pathogenic
GCK	NM_000162.5	c.128G>C	p.R43P	Likely pathogenic
GCK	NM_000162.5	c.128G>T	p.R43L	Likely pathogenic
GCK	NM_000162.5	c.1292C>A	p.T431K	Likely pathogenic
GCK	NM_000162.5	c.1300T>G	p.C434G	Likely pathogenic
GCK	NM_000162.5	c.1306A>T	p.I436F	Likely pathogenic
GCK	NM_000162.5	c.130G>A	p.G44S	Pathogenic
GCK	NM_000162.5	c.1318G>T	p.E440*	Pathogenic
GCK	NM_000162.5	c.1319_1323dup	p.E442fs	Pathogenic
GCK	NM_000162.5	c.131del	p.G44fs	Pathogenic
GCK	NM_000162.5	c.131G>A	p.G44D	Likely pathogenic
GCK	NM_000162.5	c.1322C>A	p.S441*	Pathogenic
GCK	NM_000162.5	c.1335_1345del	p.R447fs	Pathogenic
GCK	NM_000162.5	c.1340G>A	p.R447Q	Pathogenic
GCK	NM_000162.5	c.1343del	p.G448fs	Pathogenic
GCK	NM_000162.5	c.1344del	p.A449fs	Pathogenic
GCK	NM_000162.5	c.1346C>A	p.A449E	Pathogenic
GCK	NM_000162.5	c.1358C>G	p.S453W	Pathogenic
GCK	NM_000162.5	c.1358C>T	p.S453L	Pathogenic
GCK	NM_000162.5	c.1360del	p.A454fs	Pathogenic
GCK	NM_000162.5	c.1360G>A	p.A454T	Pathogenic
GCK	NM_000162.5	c.1361C>T	p.A454V	Likely pathogenic
GCK	NM_000162.5	c.1364T>A	p.V455E	Likely pathogenic
GCK	NM_000162.5	c.1398A>T	p.*466Cext*144	Pathogenic
GCK	NM_000162.5	c.148C>T	p.H50Y	Likely pathogenic
GCK	NM_000162.5	c.149A>T	p.H50L	Likely pathogenic
GCK	NM_000162.5	c.158C>T	p.A53V	Pathogenic
GCK	NM_000162.5	c.162T>G	p.S54R	Likely pathogenic

GCK	NM_000162.5	c.171G>A	p.M57I	Pathogenic
GCK	NM_000162.5	c.183C>A	p.Y61*	Pathogenic
GCK	NM_000162.5	c.183C>G	p.Y61*	Pathogenic
GCK	NM_000162.5	c.184G>A	p.V62M	Pathogenic
GCK	NM_000162.5	c.208+3A>G	p.?	Pathogenic
GCK	NM_000162.5	c.208G>C	p.E70Q	Likely pathogenic
GCK	NM_000162.5	c.214G>A	p.G72R	Pathogenic
GCK	NM_000162.5	c.214G>C	p.G72R	Pathogenic
GCK	NM_000162.5	c.217del	p.D73fs	Pathogenic
GCK	NM_000162.5	c.227C>T	p.S76F	Likely pathogenic
GCK	NM_000162.5	c.232G>A	p.D78N	Pathogenic
GCK	NM_000162.5	c.232G>C	p.D78H	Pathogenic
GCK	NM_000162.5	c.233A>G	p.D78G	Likely pathogenic
GCK	NM_000162.5	c.234C>G	p.D78E	Pathogenic
GCK	NM_000162.5	c.236T>C	p.L79P	Likely pathogenic
GCK	NM_000162.5	c.238G>A	p.G80S	Likely pathogenic
GCK	NM_000162.5	c.238G>C	p.G80R	Pathogenic
GCK	NM_000162.5	c.239G>T	p.G80V	Pathogenic
GCK	NM_000162.5	c.241G>A	p.G81S	Pathogenic
GCK	NM_000162.5	c.256_258del	p.V86del	Pathogenic
GCK	NM_000162.5	c.261G>T	p.M87I	Likely pathogenic
GCK	NM_000162.5	c.297G>A	p.W99*	Pathogenic
GCK	NM_000162.5	c.298del	p.S100fs	Pathogenic
GCK	NM_000162.5	c.301del	p.V101*	Pathogenic
GCK	NM_000162.5	c.313_314insGGAGACCAAAC	p.H105fs	Pathogenic
GCK	NM_000162.5	c.316C>T	p.Q106*	Pathogenic
GCK	NM_000162.5	c.320T>G	p.M107R	Likely pathogenic
GCK	NM_000162.5	c.322T>C	p.Y108H	Pathogenic
GCK	NM_000162.5	c.324C>A	p.Y108*	Pathogenic
GCK	NM_000162.5	c.325T>C	p.S109P	Likely pathogenic
GCK	NM_000162.5	c.332C>T	p.P111L	Pathogenic
GCK	NM_000162.5	c.351_358del	p.T118fs	Pathogenic
GCK	NM_000162.5	c.353C>T	p.T118I	Likely pathogenic
GCK	NM_000162.5	c.364C>A	p.L122I	Likely pathogenic
GCK	NM_000162.5	c.367_369del	p.F123del	Pathogenic
GCK	NM_000162.5	c.370G>A	p.D124N	Pathogenic
GCK	NM_000162.5	c.386G>A	p.C129Y	Likely pathogenic
GCK	NM_000162.5	c.389T>C	p.I130T	Likely pathogenic
GCK	NM_000162.5	c.391T>C	p.S131P	Likely pathogenic
GCK	NM_000162.5	c.409C>G	p.H137D	Likely pathogenic
GCK	NM_000162.5	c.435_436dup	p.L146fs	Pathogenic
GCK	NM_000162.5	c.448T>G	p.F150V	Pathogenic
GCK	NM_000162.5	c.449T>C	p.F150S	Pathogenic
GCK	NM_000162.5	c.45+1G>T	p.?	Pathogenic
GCK	NM_000162.5	c.45+3A>G	p.?	Likely pathogenic
GCK	NM_000162.5	c.451T>C	p.S151P	Likely pathogenic

GCK	NM_000162.5	c.452_454del	p.S151del	Pathogenic
GCK	NM_000162.5	c.458C>A	p.P153H	Likely pathogenic
GCK	NM_000162.5	c.46-?_363+?del	p.?	Pathogenic
GCK	NM_000162.5	c.461T>C	p.V154A	Likely pathogenic
GCK	NM_000162.5	c.466C>G	p.H156D	Likely pathogenic
GCK	NM_000162.5	c.466C>T	p.H156Y	Likely pathogenic
GCK	NM_000162.5	c.469G>A	p.E157K	Likely pathogenic
GCK	NM_000162.5	c.475A>G	p.I159V	Likely pathogenic
GCK	NM_000162.5	c.475A>T	p.I159F	Likely pathogenic
GCK	NM_000162.5	c.476T>A	p.I159N	Likely pathogenic
GCK	NM_000162.5	c.478G>A	p.D160N	Pathogenic
GCK	NM_000162.5	c.483+1G>A	p.?	Pathogenic
GCK	NM_000162.5	c.483+2_483+16del	p.?	Pathogenic
GCK	NM_000162.5	c.484-?_679+?del	p.?	Pathogenic
GCK	NM_000162.5	c.485G>A	p.G162D	Likely pathogenic
GCK	NM_000162.5	c.488T>A	p.I163N	Likely pathogenic
GCK	NM_000162.5	c.491T>C	p.L164P	Pathogenic
GCK	NM_000162.5	c.500G>A	p.W167*	Pathogenic
GCK	NM_000162.5	c.508G>A	p.G170S	Likely pathogenic
GCK	NM_000162.5	c.511T>C	p.F171L	Pathogenic
GCK	NM_000162.5	c.521C>G	p.S174*	Pathogenic
GCK	NM_000162.5	c.523G>C	p.G175R	Pathogenic
GCK	NM_000162.5	c.524G>A	p.G175E	Likely pathogenic
GCK	NM_000162.5	c.527C>A	p.A176E	Pathogenic
GCK	NM_000162.5	c.533G>A	p.G178E	Pathogenic
GCK	NM_000162.5	c.533G>C	p.G178A	Pathogenic
GCK	NM_000162.5	c.533G>T	p.G178V	Likely pathogenic
GCK	NM_000162.5	c.539A>G	p.N180S	Pathogenic
GCK	NM_000162.5	c.540T>G	p.N180K	Pathogenic
GCK	NM_000162.5	c.544G>A	p.V182M	Pathogenic
GCK	NM_000162.5	c.544G>C	p.V182L	Pathogenic
GCK	NM_000162.5	c.554T>C	p.L185P	Likely pathogenic
GCK	NM_000162.5	c.556C>T	p.R186*	Pathogenic
GCK	NM_000162.5	c.557G>C	p.R186P	Pathogenic
GCK	NM_000162.5	c.-557G>C	p.?	Pathogenic
GCK	NM_000162.5	c.562G>A	p.A188T	Pathogenic
GCK	NM_000162.5	c.563C>G	p.A188G	Likely pathogenic
GCK	NM_000162.5	c.567_568insTATC	p.K190fs	Pathogenic
GCK	NM_000162.5	c.571C>T	p.R191W	Pathogenic
GCK	NM_000162.5	c.572G>A	p.R191Q	Pathogenic
GCK	NM_000162.5	c.579+1G>A	p.?	Pathogenic
GCK	NM_000162.5	c.579G>T	p.?	Likely pathogenic
GCK	NM_000162.5	c.579G>T	p.G193G	Likely pathogenic
GCK	NM_000162.5	c.580-1G>A	p.?	Pathogenic
GCK	NM_000162.5	c.580del	p.D194fs	Pathogenic
GCK	NM_000162.5	c.596T>C	p.V199A	Likely pathogenic

GCK	NM_000162.5	c.59T>C	p.L20P	Likely pathogenic
GCK	NM_000162.5	c.601G>T	p.A201S	Pathogenic
GCK	NM_000162.5	c.605T>C	p.M202T	Pathogenic
GCK	NM_000162.5	c.605T>G	p.M202R	Pathogenic
GCK	NM_000162.5	c.608T>C	p.V203A	Pathogenic
GCK	NM_000162.5	c.614dup	p.D205fs	Pathogenic
GCK	NM_000162.5	c.616A>C	p.T206P	Pathogenic
GCK	NM_000162.5	c.617C>T	p.T206M	Pathogenic
GCK	NM_000162.5	c.623C>T	p.A208V	Pathogenic
GCK	NM_000162.5	c.626C>T	p.T209M	Pathogenic
GCK	NM_000162.5	c.637T>C	p.C213R	Pathogenic
GCK	NM_000162.5	c.641dup	p.Y214*	Pathogenic
GCK	NM_000162.5	c.645C>A	p.Y215*	Pathogenic
GCK	NM_000162.5	c.645C>G	p.Y215*	Pathogenic
GCK	NM_000162.5	c.655C>T	p.Q219*	Pathogenic
GCK	NM_000162.5	c.660C>A	p.C220*	Pathogenic
GCK	NM_000162.5	c.661G>A	p.E221K	Pathogenic
GCK	NM_000162.5	c.667G>A	p.G223S	Pathogenic
GCK	NM_000162.5	c.675C>G	p.I225M	Pathogenic
GCK	NM_000162.5	c.676G>A	p.V226M	Pathogenic
GCK	NM_000162.5	c.679+1G>A	p.?	Pathogenic
GCK	NM_000162.5	c.679+2T>C	p.?	Likely pathogenic
GCK	NM_000162.5	c.679G>C	p.G227R	Likely pathogenic
GCK	NM_000162.5	c.67T>G	p.F23V	Pathogenic
GCK	NM_000162.5	c.680-15C>A	p.?	Likely pathogenic
GCK	NM_000162.5	c.680-2A>G	p.?	Pathogenic
GCK	NM_000162.5	c.683C>A	p.T228K	Pathogenic
GCK	NM_000162.5	c.683C>T	p.T228M	Pathogenic
GCK	NM_000162.5	c.685G>A	p.G229S	Likely pathogenic
GCK	NM_000162.5	c.686G>T	p.G229V	Likely pathogenic
GCK	NM_000162.5	c.688T>G	p.C230G	Likely pathogenic
GCK	NM_000162.5	c.703A>G	p.M235V	Pathogenic
GCK	NM_000162.5	c.704T>A	p.M235K	Pathogenic
GCK	NM_000162.5	c.704T>C	p.M235T	Pathogenic
GCK	NM_000162.5	c.706G>A	p.E236K	Pathogenic
GCK	NM_000162.5	c.713T>A	p.M238K	Likely pathogenic
GCK	NM_000162.5	c.722T>C	p.V241A	Likely pathogenic
GCK	NM_000162.5	c.728T>C	p.L243P	Pathogenic
GCK	NM_000162.5	c.731T>A	p.V244E	Likely pathogenic
GCK	NM_000162.5	c.737G>A	p.G246E	Likely pathogenic
GCK	NM_000162.5	c.739del	p.D247fs	Pathogenic
GCK	NM_000162.5	c.740_740delinsGC	p.D247fs	Pathogenic
GCK	NM_000162.5	c.745G>C	p.G249R	Likely pathogenic
GCK	NM_000162.5	c.748C>T	p.R250C	Pathogenic
GCK	NM_000162.5	c.751A>G	p.M251V	Pathogenic
GCK	NM_000162.5	c.752T>C	p.M251T	Likely pathogenic

GCK	NM_000162.5	c.753G>A	p.M251I	Likely pathogenic
GCK	NM_000162.5	c.757del	p.V253fs	Pathogenic
GCK	NM_000162.5	c.760A>C	p.N254H	Likely pathogenic
GCK	NM_000162.5	c.766G>A	p.E256K	Pathogenic
GCK	NM_000162.5	c.769dup	p.W257fs	Pathogenic
GCK	NM_000162.5	c.76C>T	p.Q26*	Pathogenic
GCK	NM_000162.5	c.770G>A	p.W257*	Pathogenic
GCK	NM_000162.5	c.771G>C	p.W257C	Likely pathogenic
GCK	NM_000162.5	c.772G>A	p.G258S	Likely pathogenic
GCK	NM_000162.5	c.773G>A	p.G258D	Likely pathogenic
GCK	NM_000162.5	c.775G>A	p.A259T	Pathogenic
GCK	NM_000162.5	c.776C>T	p.A259V	Pathogenic
GCK	NM_000162.5	c.778T>G	p.F260V	Likely pathogenic
GCK	NM_000162.5	c.781G>A	p.G261R	Pathogenic
GCK	NM_000162.5	c.781G>C	p.G261R	Pathogenic
GCK	NM_000162.5	c.784del	p.D262fs	Pathogenic
GCK	NM_000162.5	c.787T>C	p.S263P	Pathogenic
GCK	NM_000162.5	c.790G>A	p.G264S	Pathogenic
GCK	NM_000162.5	c.793G>A	p.E265K	Pathogenic
GCK	NM_000162.5	c.79G>T	p.E27*	Pathogenic
GCK	NM_000162.5	c.802G>A	p.E268K	Pathogenic
GCK	NM_000162.5	c.820G>A	p.D274N	Likely pathogenic
GCK	NM_000162.5	c.823C>T	p.R275C	Pathogenic
GCK	NM_000162.5	c.824G>C	p.R275P	Likely pathogenic
GCK	NM_000162.5	c.829_830insCGG	p.L276_V277insA	Pathogenic
GCK	NM_000162.5	c.834C>G	p.D278E	Pathogenic
GCK	NM_000162.5	c.835G>T	p.E279*	Pathogenic
GCK	NM_000162.5	c.852C>G	p.P284P	Pathogenic
GCK	NM_000162.5	c.852del	p.G285fs	Pathogenic
GCK	NM_000162.5	c.854G>A	p.G285D	Pathogenic
GCK	NM_000162.5	c.856C>T	p.Q286*	Pathogenic
GCK	NM_000162.5	c.860A>C	p.Q287P	Pathogenic
GCK	NM_000162.5	c.864-1G>A	p.?	Pathogenic
GCK	NM_000162.5	c.865T>C	p.Y289H	Likely pathogenic
GCK	NM_000162.5	c.867T>G	p.Y289*	Pathogenic
GCK	NM_000162.5	c.870G>T	p.E290D	Likely pathogenic
GCK	NM_000162.5	c.877A>C	p.I293L	Pathogenic
GCK	NM_000162.5	c.878T>C	p.I293T	Likely pathogenic
GCK	NM_000162.5	c.878T>G	p.I293R	Pathogenic
GCK	NM_000162.5	c.884_885delinsTT	p.G295V	Pathogenic
GCK	NM_000162.5	c.884del	p.G295fs	Pathogenic
GCK	NM_000162.5	c.885_895del	p.K296fs	Pathogenic
GCK	NM_000162.5	c.895G>C	p.G299R	Pathogenic
GCK	NM_000162.5	c.896del	p.G299fs	Pathogenic
GCK	NM_000162.5	c.896G>A	p.G299D	Pathogenic
GCK	NM_000162.5	c.896G>T	p.G299V	Pathogenic

GCK	NM_000162.5	c.898G>A	p.E300K	Likely pathogenic
GCK	NM_000162.5	c.904G>C	p.V302L	Pathogenic
GCK	NM_000162.5	c.907C>T	p.R303W	Likely pathogenic
GCK	NM_000162.5	c.908G>T	p.R303L	Pathogenic
GCK	NM_000162.5	c.911T>C	p.L304P	Pathogenic
GCK	NM_000162.5	c.912_915dup	p.L306fs	Pathogenic
GCK	NM_000162.5	c.917T>C	p.L306P	Likely pathogenic
GCK	NM_000162.5	c.920T>C	p.L307P	Likely pathogenic
GCK	NM_000162.5	c.925_953del	p.L309fs	Pathogenic
GCK	NM_000162.5	c.929T>G	p.V310G	Likely pathogenic
GCK	NM_000162.5	c.934_937dup	p.D311fs	Pathogenic
GCK	NM_000162.5	c.938del	p.N313fs	Pathogenic
GCK	NM_000162.5	c.941T>C	p.L314P	Likely pathogenic
GCK	NM_000162.5	c.941T>G	p.L314R	Likely pathogenic
GCK	NM_000162.5	c.946T>G	p.F316V	Likely pathogenic
GCK	NM_000162.5	c.951C>G	p.H317Q	Likely pathogenic
GCK	NM_000162.5	c.952G>A	p.G318R	Pathogenic
GCK	NM_000162.5	c.971T>C	p.L324P	Pathogenic
GCK	NM_000162.5	c.98T>C	p.V33A	Likely pathogenic
GCK	NM_000162.5	c.995C>A	p.T332K	Likely pathogenic
GCK	NM_000162.5	c.995C>G	p.T332R	Likely pathogenic
GCK	NM_000162.5	g.(44,193,063_44,228,495)_(44,228,995_?)del	p.0?	Pathogenic

ESM Table 3: Pathogenic *GCK* variants identified in the clinically referred cohort.

Gene	Transcript	DNA nomenclature	Protein nomenclature	Classification
GCK	NM_000162.5	c.1358C>T	p.Ser453Leu	Likely Pathogenic
GCK	NM_000162.5	c.1346C>T	p.Ala449Val	Likely Pathogenic
GCK	NM_000162.5	c.1322C>T	p.Ser441Leu	Likely Pathogenic
GCK	NM_000162.5	c.1306A>T	p.Ile436Phe	Likely Pathogenic
GCK	NM_000162.5	c.1264C>T	p.Arg422Trp	Likely Pathogenic
GCK	NM_000162.5	c.1228G>C	p.Gly410Arg	Likely Pathogenic
GCK	NM_000162.5	c.1174C>T	p.Arg392Cys	Pathogenic
GCK	NM_000162.5	c.1148C>T	p.Ser383Leu	Likely Pathogenic
GCK	NM_000162.5	c.1142T>C	p.Met381Thr	Pathogenic
GCK	NM_000162.5	c.1132G>A	p.Ala378Thr	Likely Pathogenic
GCK	NM_000162.5	c.1019+2T>C	p.?	Pathogenic
GCK	NM_000162.5	c.1019G>T	p.Ser340Ile	Pathogenic
GCK	NM_000162.5	c.951C>G	p.His317Gln	Likely Pathogenic
GCK	NM_000162.5	c.902T>C	p.Leu301Pro	Likely Pathogenic
GCK	NM_000162.5	c.878T>G	p.Ile293Arg	Likely Pathogenic
GCK	NM_000162.5	c.878T>C	p.Ile293Thr	Likely Pathogenic
GCK	NM_000162.5	c.864-1G>A	p.?	Pathogenic
GCK	NM_000162.5	c.835G>T	p.Glu279*	Pathogenic
GCK	NM_000162.5	c.823C>G	p.Arg275Gly	Likely Pathogenic
GCK	NM_000162.5	c.793G>A	p.Glu265Lys	Pathogenic
GCK	NM_000162.5	c.766G>A	p.Glu256Lys	Pathogenic
GCK	NM_000162.5	c.748C>T	p.Arg250Cys	Pathogenic
GCK	NM_000162.5	c.676G>A	p.Val226Met	Pathogenic
GCK	NM_000162.5	c.667G>A	p.Gly223Ser	Pathogenic
GCK	NM_000162.5	c.661G>A	p.Glu221Lys	Pathogenic
GCK	NM_000162.5	c.660C>A	p.Cys220*	Pathogenic
GCK	NM_000162.5	c.645C>G	p.Tyr215*	Pathogenic
GCK	NM_000162.5	c.645C>A	p.Tyr215*	Pathogenic
GCK	NM_000162.5	c.641dup	p.Tyr214*	Pathogenic
GCK	NM_000162.5	c.626C>T	p.Thr209Met	Likely Pathogenic
GCK	NM_000162.5	c.601G>T	p.Ala201Ser	Likely Pathogenic
GCK	NM_000162.5	c.580-13_580-1del	p.?	Pathogenic
GCK	NM_000162.5	c.580-1G>A	p.?	Pathogenic
GCK	NM_000162.5	c.579+1G>A	p.?	Pathogenic
GCK	NM_000162.5	c.571C>T	p.Arg191Trp	Pathogenic
GCK	NM_000162.5	c.562G>A	p.Ala188Thr	Pathogenic
GCK	NM_000162.5	c.556C>T	p.Arg186*	Pathogenic
GCK	NM_000162.5	c.544G>A	p.Val182Met	Pathogenic
GCK	NM_000162.5	c.540T>G	p.Asn180Lys	Pathogenic
GCK	NM_000162.5	c.478G>T	p.Asp160Tyr	Likely Pathogenic
GCK	NM_000162.5	c.478G>A	p.Asp160Asn	Pathogenic
GCK	NM_000162.5	c.461T>C	p.Val154Ala	Likely Pathogenic
GCK	NM_000162.5	c.391T>C	p.Ser131Pro	Likely Pathogenic

GCK	NM_000162.5	c.386G>A	p.Cys129Tyr	Likely Pathogenic
GCK	NM_000162.5	c.370G>A	p.Asp124Asn	Pathogenic
GCK	NM_000162.5	c.363+2del	p.?	Pathogenic
GCK	NM_000162.5	c.351_358del	p.Thr118Aspfs*8	Pathogenic
GCK	NM_000162.5	c.316C>T	p.Gln106*	Pathogenic
GCK	NM_000162.5	c.209-1G>A	p.?	Pathogenic
GCK	NM_000162.5	c.208+2T>C	p.?	Pathogenic
GCK	NM_000162.5	c.184G>A	p.Val62Met	Likely Pathogenic

ESM Table 4: Pathogenic *GCK* variants identified in the UK Biobank.

Trait	PubMed ID	N SNPs in Score	Comments
Type 2 Diabetes (T2D)	38374256	1289	Constructed using plink – score function using genome wide significant variants
Type 1 Diabetes (T1D)	30655379	67	Weighted T1D score using T1DGRS2, available at: https://github.com/t2diabetesgenes/t1dgrs2
Acute Insulin Response	28490609	955764	Genome-wide polygenic scores, we implemented the GenoPred 2.2.1 pipeline with LDpred2's auto model, which included quality control of summary statistics and genetic data
Body Mass Index (BMI)	25673413	886707	
Fasting Insulin	34059833	1036765	
Waist Hip Ratio (WHR)	30239722	906879	
Fasting Glucose	34059833	110<br>(67 T2D, 34 non-T2D)	Constructed using plink – score function using genome wide significant variants
HbA_{1c}	34059833	132<br>(57 T2D, 54 non T2D)<br>(35 Glycaemic, 75 non-Glycaemic)	
Lipodystrophy	27841877	53	
Type 2 Diabetes (T2D)*	39379762	1087858	Genome-wide polygenic score for T2D, using weights previously derived using PRS-CS, which excluding UKBB participants during testing

ESM Table 5: Polygenic Scores used in analysis. Polygenic scores (PGS) for 9 diabetes related traits used in the analysis. For each trait, the corresponding PubMed ID, and the number of SNPs included in the score are provided. * Used for UK Biobank analysis as it does contain have weights from UK Biobank.

Predictor	Effect Size (change in HbA1c, mmol/mol) (95% CI)	*P*
HbA_{1c} PGS (per SD increase)	0.78 (0.48 – 1.09)	4.87×10−7
Sex (male vs females)	-1.84 (-2.67 – -1.01)	1.64×10−5
BMI (per kg/m^2 increase)	0.12 (0.03 – 0.21)	0.007
Age (per year increase)	0.003 (-0.02 – 0.02)	0.81
Mutation Type (Missense vs PTVs)	1.04 (0.11- 1.97)	0.02
Parent Diabetes History		
Mother	-1.39 (-2.48 – -0.31)	0.01
Father	-1.08 (-2.11 – -0.05)	0.03
Both	-1.64 (-3.94 – 0.64)	0.15

ESM Table 6: HbA_{1c} PGS increases HbA_{1c} in GCK-MODY cases even after adjusting for clinical and genotype characteristics. Hba1c PGS was included in a mixed effect linear regression model with HbA_{1c} (mmol/mol) as the outcome, and family ID as the random effect. Other covariates included in the model: Sex, Age, BMI, Parental History, Mutation Type and 10 genetic ancestry principal components. All effect sizes in are in DCCT units. Parental Diabetes in reference to subjects whose parents had no history of diabetes. Missense variants, Males and Individuals with no parental history are the reference group in their respective predictor. Protein-truncating variants = PTVs.

Predictor	OR (95 CI) Local Cohort	*P* Local Cohort	OR (95 CI) UKBB	*P* UKBB
HbA_{1c} PGS Quintile				
Middle 60%	1.89 (1.2 – 2.99)	0.006	2.04 (0.81 – 5.11)	0.12
Top 20%	2.79 (1.67 - 4.68)	1.2×10^{-4}	5.34 (1.65 – 17.27)	0.005
HbA_{1c} PGS Quintile (Adjusted)				
Middle 60%	2.17 (1.25 -3.76)	0.006	2.36 (0.84 -6.62)	0.1
Top 20%	3.08 (1.66 -5.76)	4.2×10^{-4}	6.06 (1.59 – 23.03)	0.008

ESM Table 7: Increased HbA_{1c} PGS associated with higher likelihood of exceeding diagnostic diabetes threshold. The likelihood of having an HbA1c level ≥48 mmol/mol in *GCK* carriers, was assessed by stratifying individuals into HbA_{1c} PGS quintiles (bottom 20%, middle 60%, and top 20%). In the local cohort, unadjusted and adjusted mixed-effects logistic models were used, with family-id as a random effect. In the UK Biobank (UKBB), standard logistic regression was applied. Adjusted models included the following covariates, local cohort: sex, age, BMI, parental history of diabetes, mutation type, year of diabetes diagnosis, and 10 genetic ancestry principal components, UKBB: sex, age, BMI, parental history of diabetes, and mutation type, genetic principial components. Odds ratios and 95% confidence intervals are shown for the middle and top quintiles, compared to the bottom quintile.

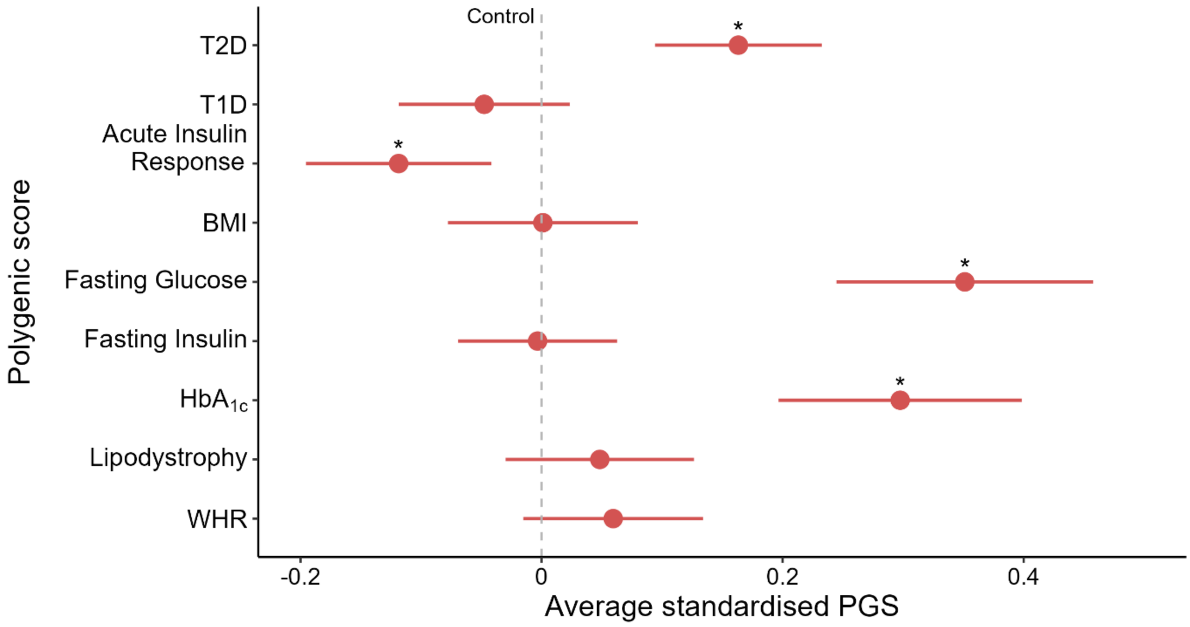

ESM Figure 1: Polygenic Risk in clinically referred GCK-MODY probands.

Standardised difference in nine diabetes related polygenic scores, each assed separately using linear regression. GCK-MODY probands (red, N= 705), are compared against control individuals without diabetes (dashed grey line, N = 7,645). All scores are standardised to have a mean of 0 and standard deviation of 1 in controls. Asterisks denote Bonferroni-adjusted statistically significant differences from controls ($p < 0.0056$). Error bars represent 95% confidence intervals. BMI = Body Mass Index, WHR = Waist Hip Ratio.

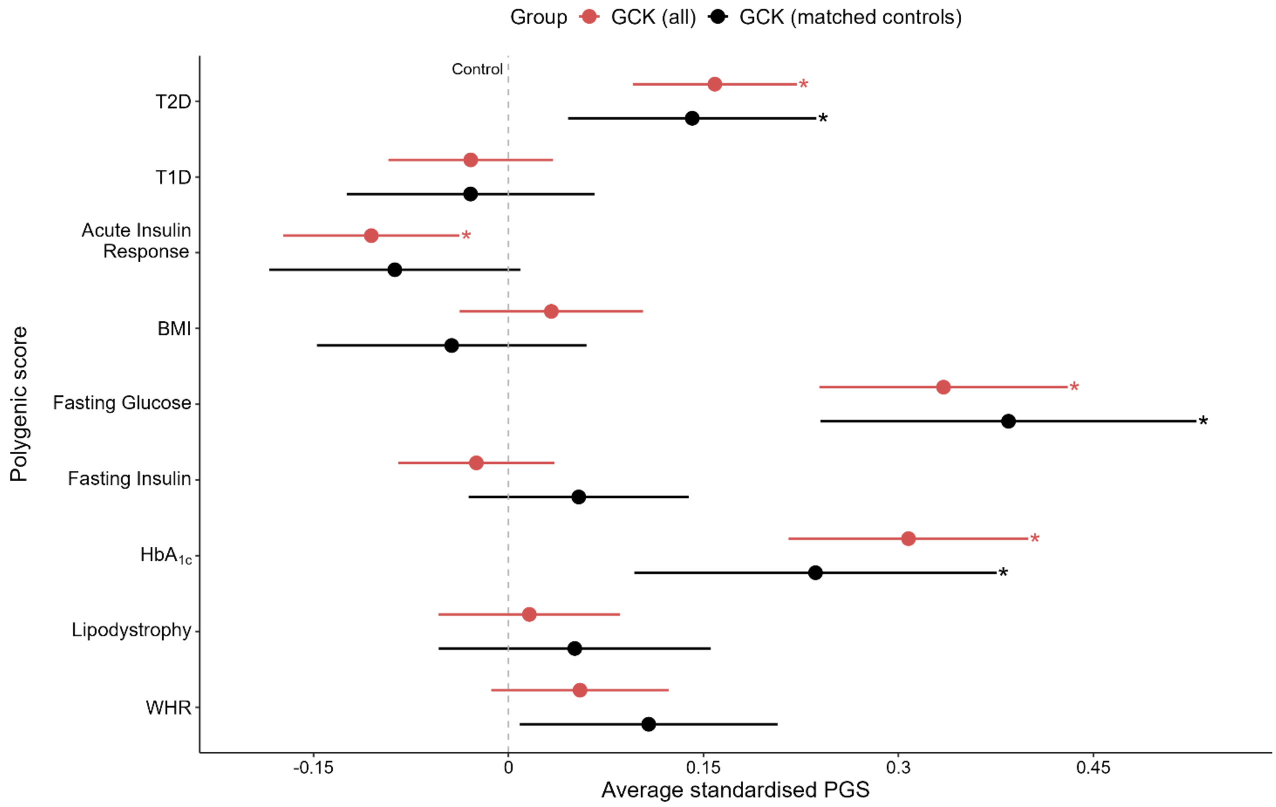

ESM Figure 2: Similar polygenic enrichment in GCK-MODY when using matched controls.

Standardised differences in nine diabetes-related polygenic scores, each assessed separately using linear regression. GCK-MODY cases (red, N = 897) are compared with unmatched non-diabetic controls (grey dashed line, N = 7,645) and with age- (±1 year) and sex-matched controls (black, N = 722 pairs). All scores were standardised to mean 0 and SD 1 in controls. Asterisks denote Bonferroni-adjusted significant differences from controls ($p < 0.0056$). Error bars show 95% confidence intervals. BMI = Body Mass Index, WHR = Waist-Hip Ratio.

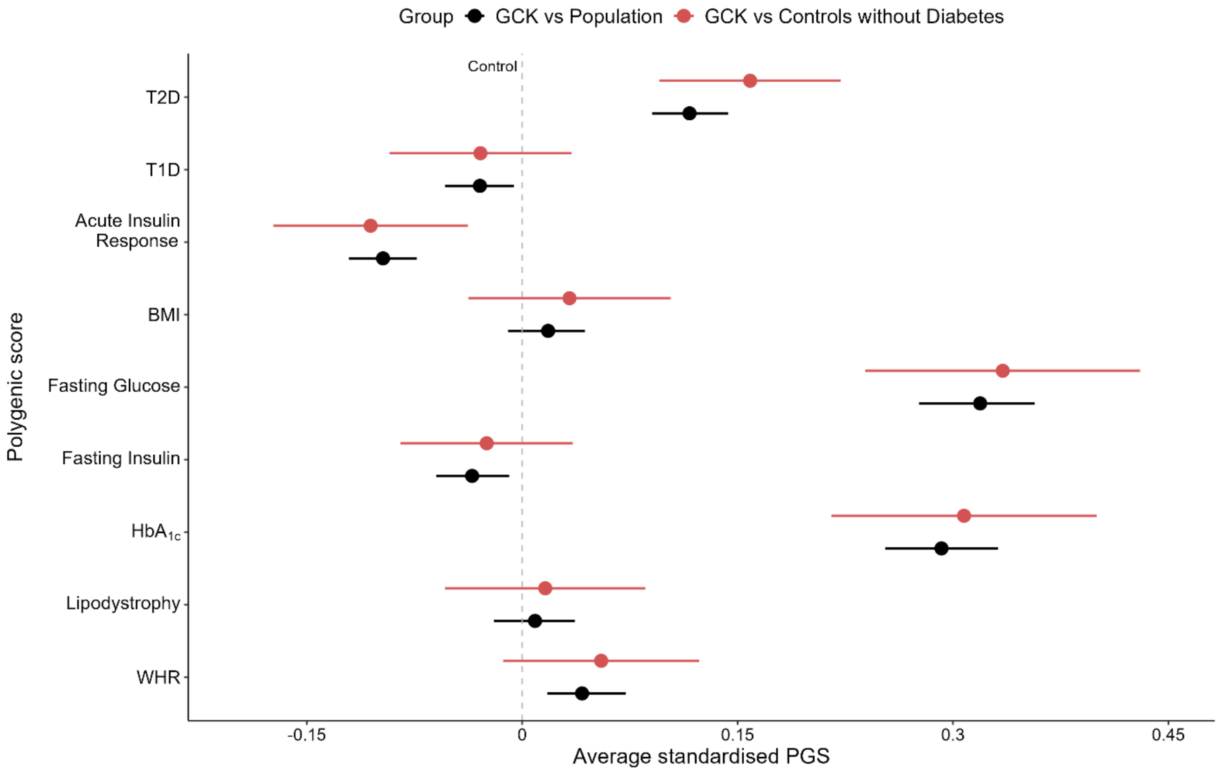

ESM Figure 3: Similar polygenic enrichment in GCK-MODY when compared with a resampled reference population including T2D cases.

Standardised differences in nine diabetes-related polygenic scores, each assessed separately using linear regression. GCK-MODY cases (red, N = 897) are compared with non-diabetic controls (N = 7,645). In black, GCK-MODY cases are compared with a bootstrap-derived reference population (500 resamples of 5,000 individuals each) constructed to include 7% T2D cases. All scores were standardised to mean 0 and SD 1 in controls. Error bars for the red estimates show 95% confidence intervals from the regression model. Error bars for the black estimates show 95% confidence intervals of the bootstrap mean difference. BMI = Body Mass Index, WHR = Waist-Hip Ratio.

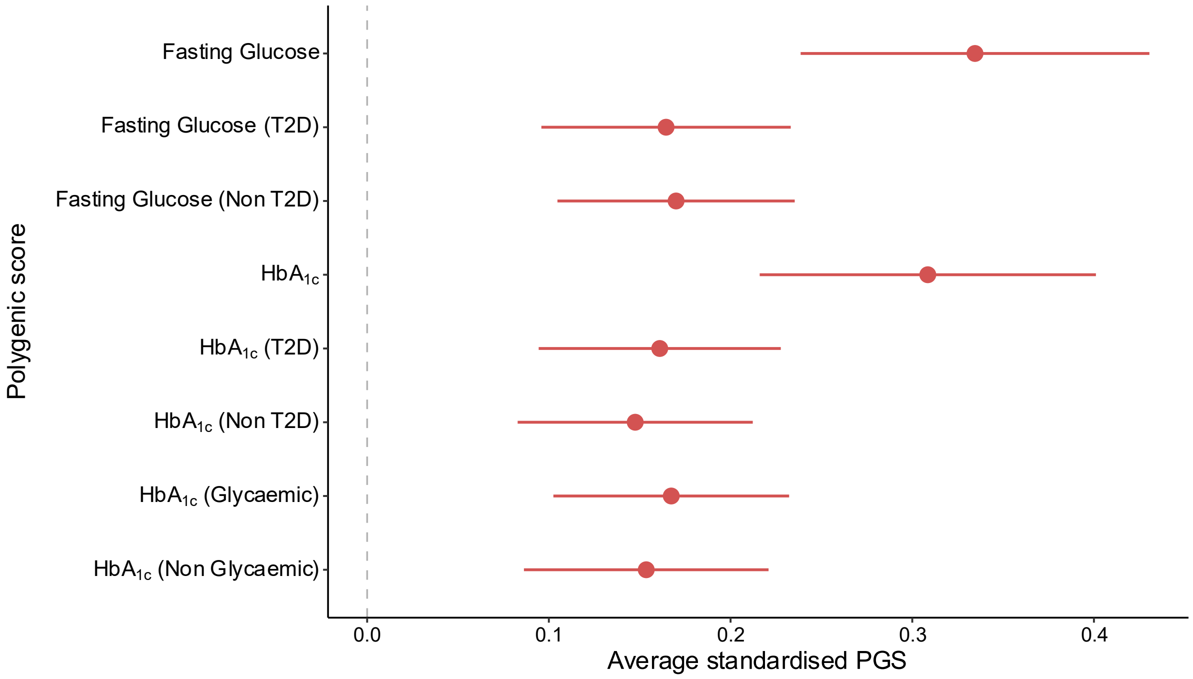

ESM Figure 4: Polygenic Risk of Partitioned HbA_{1c} and Fasting Glucose Pathways in GCK-MODY

Standardised difference in Fasting Glucose and HbA_{1c} Polygenic Scores comparing 897 GCK-MODY cases (red) versus 7,645 control individuals without diabetes (dashed grey line). Overlapping genetic variants with previous T2D association studies were identified and used to mark T2D increasing (T2D) or decreasing pathways (Non T2D). Glycaemic and non-glycaemic HbA1c variants were previously identified using signal classification, with more detail in the methods section. All scores are assessed separately using linear regression, adjusting for the first ten genetic ancestry principal components. All scores are standardised to have a mean of 0 and standard deviation of 1 in controls. Error bars represent 95% confidence intervals.

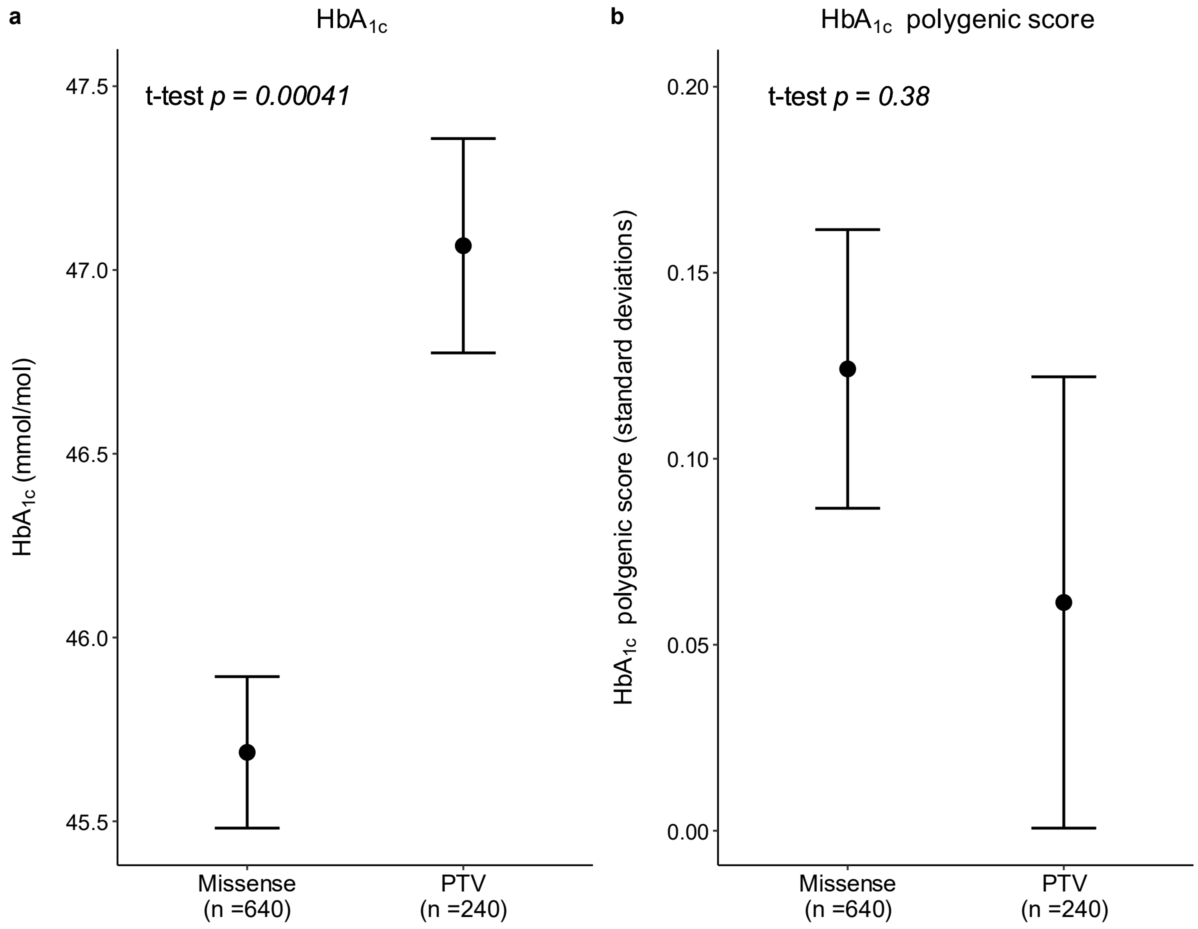

ESM Figure 5: HbA_{1c} Effects by Mutations Type in GCK-MODY.

GCK-MODY cases split by predicted effect on protein: Protein Truncting Varaint (PTV) or Missense. (a) Mean HbA_{1c} (mmol/mol) and (b) Mean HbA1c Polygenic Score (Standard Deviations). Points Represent mean values, with error bars represnting 95% confidence intervals. We assesed significance using t-tests. HbA_{1c} polygenic score was standrdised, with controls set to a mean of 0 and standard deviation of 1. Individuals with regulatory and in-frame varaints (N = 17) were excluded in this analysis.

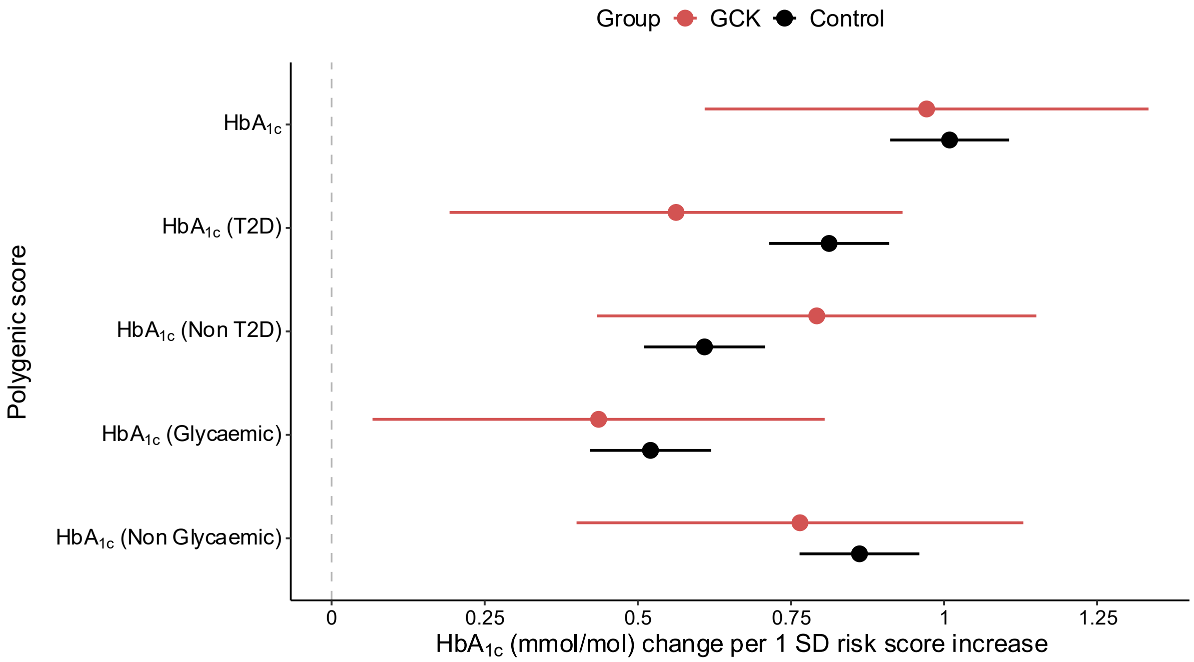

ESM Figure 6: Effect of HbA_{1c} partitioned polygenic scores on HbA_{1c} levels

Association between HbA_{1c} partitoned scores and HbA_{1c} levels. Effect sizes reperesent a mmol/mol change in HbA1c per 1 standrad deviation increase in polygenic score. All scores were assesed seperatly. In GCK-MODY (red, N = 897), estimates derived using a mixed-effects linear model with family as a random effect and adjusted for the first ten genetic ancestry principal components. For controls (black, N = 7,645), standard linear regression was used. Dots represent the estimates, with error bars indicating 95% confidence intervals. Overlapping genetic variants with previous T2D association studies were identified and used to mark T2D increasing (T2D) or decreasing pathways (Non T2D). Glycaemic and non-glycaemic HbA1c variants were previously identified using signal classification, with more detail in the methods section. All scores are standardised to have a mean of 0 and standard deviation of 1 in controls.

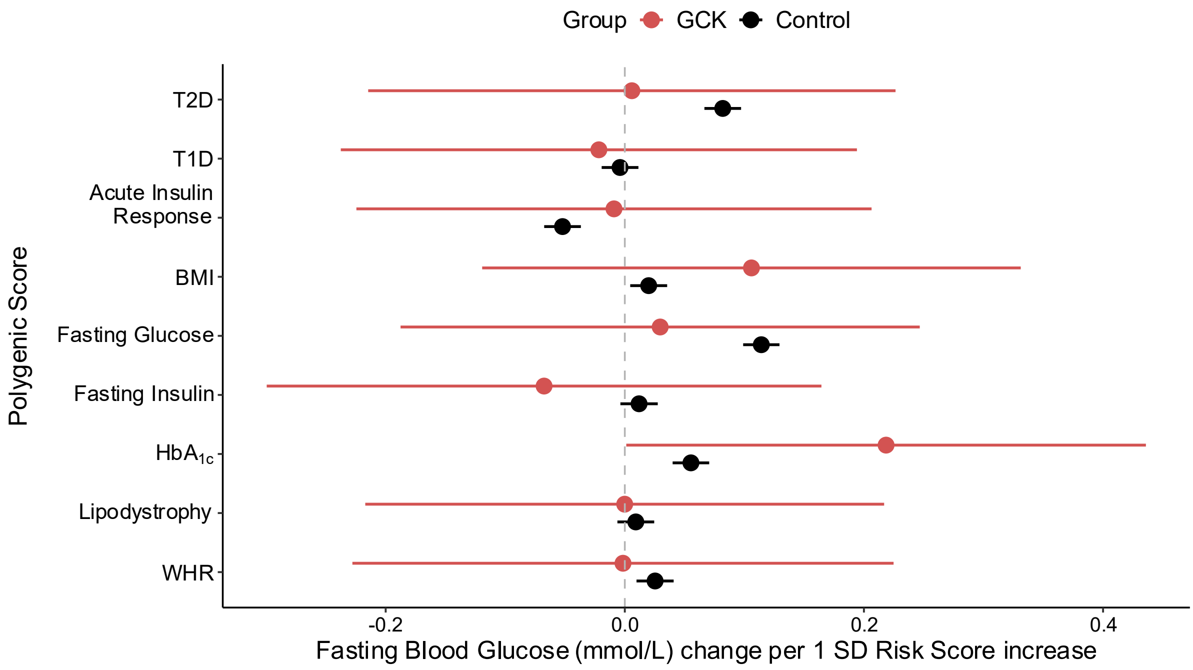

ESM Figure 7: Increased Polygenic Burden not Associted with Fasting Glucose levels in GCK-MODY.

Association between polygenic scores for nine diabetes realted traits and Fasting Glucose levels (mmol/L). All scores were assesed individually. For GCK-MODY (red, N= 897), estimates were derived using a mixed-effects linear model with family as a random effect and adjusted for the first ten genetic ancestry principal components. For controls (black, N = 7,645), standard linear regression was used. Estimates represent the effect of a 1 standard deviation increase in the respective polygenic score. Dots represent the estimates, with error bars indicating 95% confidence intervals.

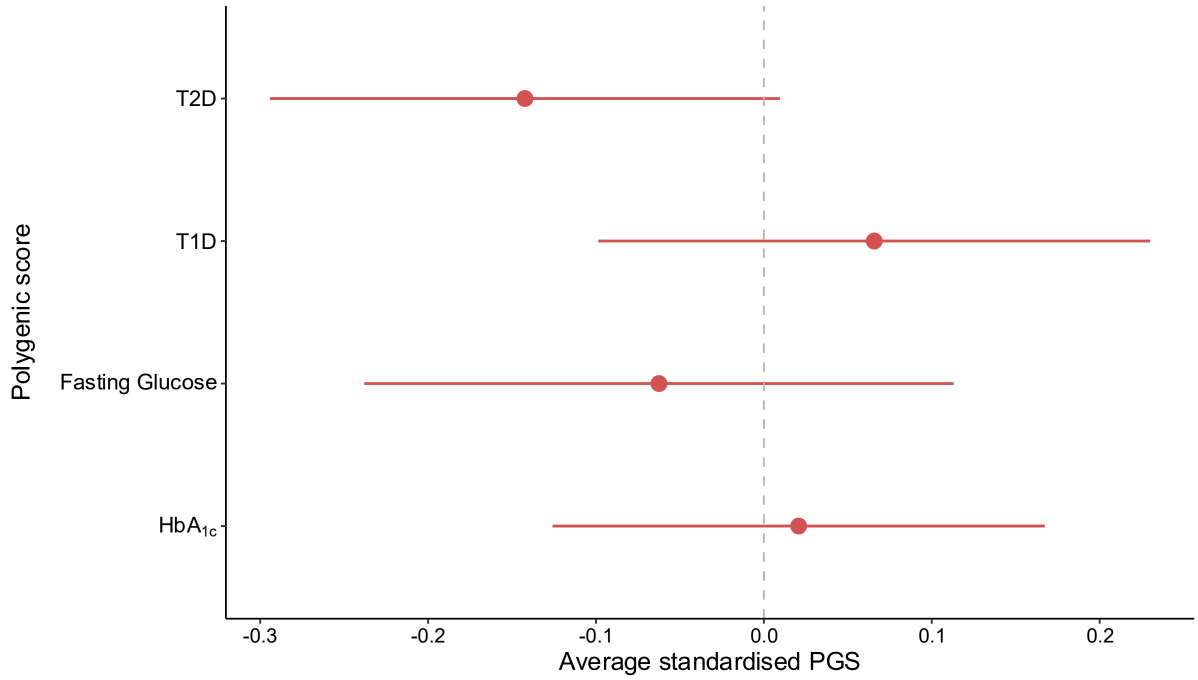

ESM Figure 8: No enrichment of polygenic background in clinically unselected *GCK* carriers

Standardized differences in four diabetes-related polygenic scores are shown. These scores were either previously identified as enriched in clinically referred GCK-MODY cases or serve as a negative control (Type 1 diabetes, T1D). Each polygenic score was evaluated separately by comparing 158 *GCK* carriers (red) to 429,333 non-carriers (dashed grey line). Linear regression was used for comparison, adjusting for the first ten principal components within the cohort. PGS = Polygenic score.

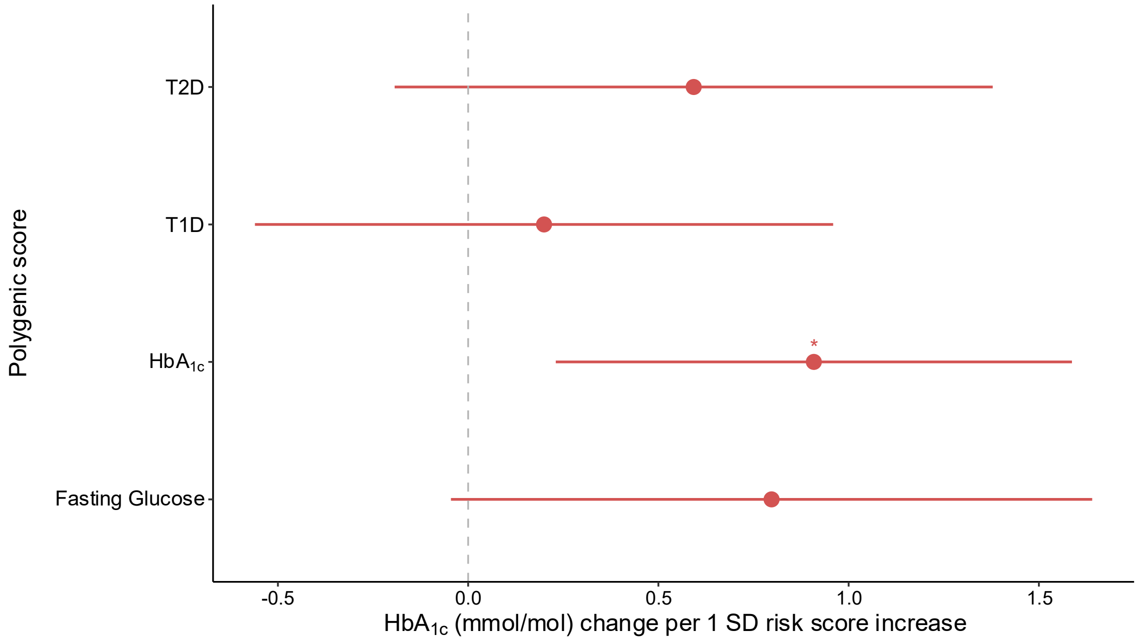

ESM Figure 9: Increased Polygenic Burden Associated With higher HbA_{1c} levels in clinically unselected *GCK* Carriers.

Association between polygenic scores for four diabetes related traits and HbA_{1c} levels (mmol/mol). These scores were either previously identified as enriched in clinically referred GCK-MODY cases or serve as a negative control (Type 1 diabetes, T1D). Each score was assessed individually in 158 *GCK* carriers (red), using linear regression models to derive estimates. Estimates represent the effect of a 1 standard deviation increase in the respective polygenic score. Dots represent the estimates, with error bars indicating 95% confidence intervals. Asterisks highlight significant differences after Bonferroni correction ($p < 0.0125$). Covariates included: Sex, Age, BMI, Parental Diabetes, Mutation Type (Protein Truncating Variant or Missense) and the first ten ancestry principal component.

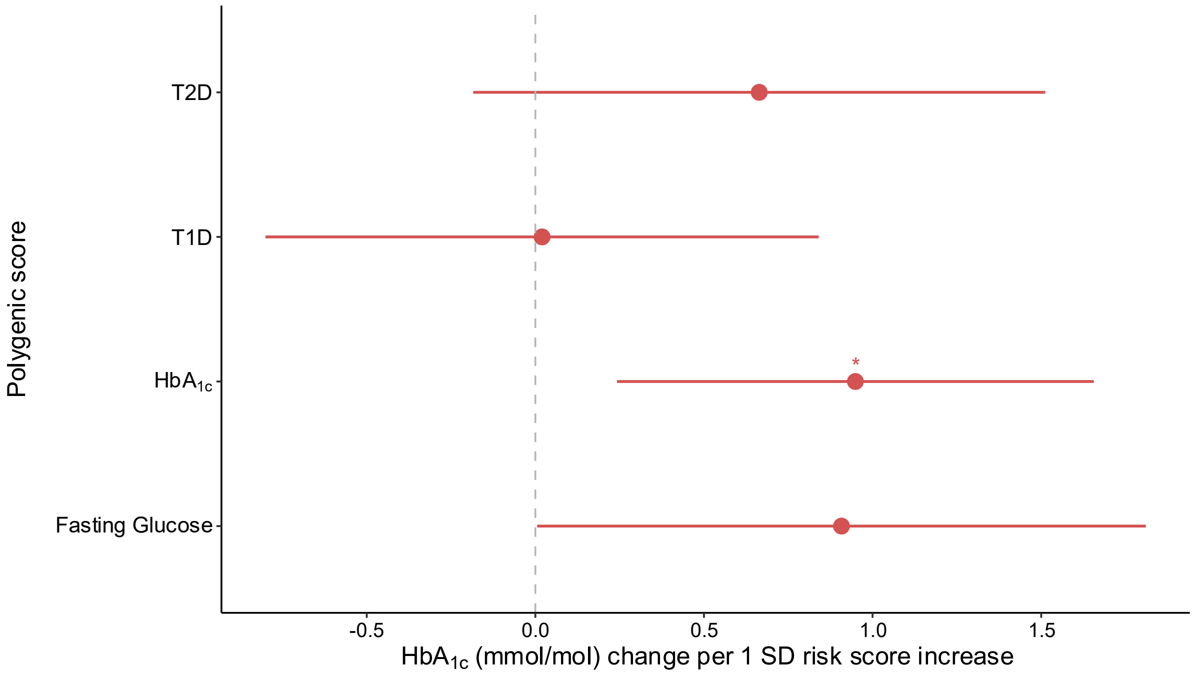

ESM Figure 10: Increased Polygenic Burden Associated With higher HbA_{1c} levels in unrelated clinically unselected GCK Carriers.

Association between polygenic scores for four diabetes related traits and HbA_{1c} levels (mmol/mol). Each score was assessed individually in 141 unrelated GCK carriers (red), using linear regression models to derive estimates. Estimates represent the effect of a 1 standard deviation increase in the respective polygenic score. Dots represent the estimates, with error bars indicating 95% confidence intervals. Asterisks highlight significant differences after Bonferroni correction ($p < 0.0125$). Covariates included: Sex, Age, BMI, Parental Diabetes, Mutation Type (Protein Truncating Variant or Missense) and the first ten ancestry principal component.

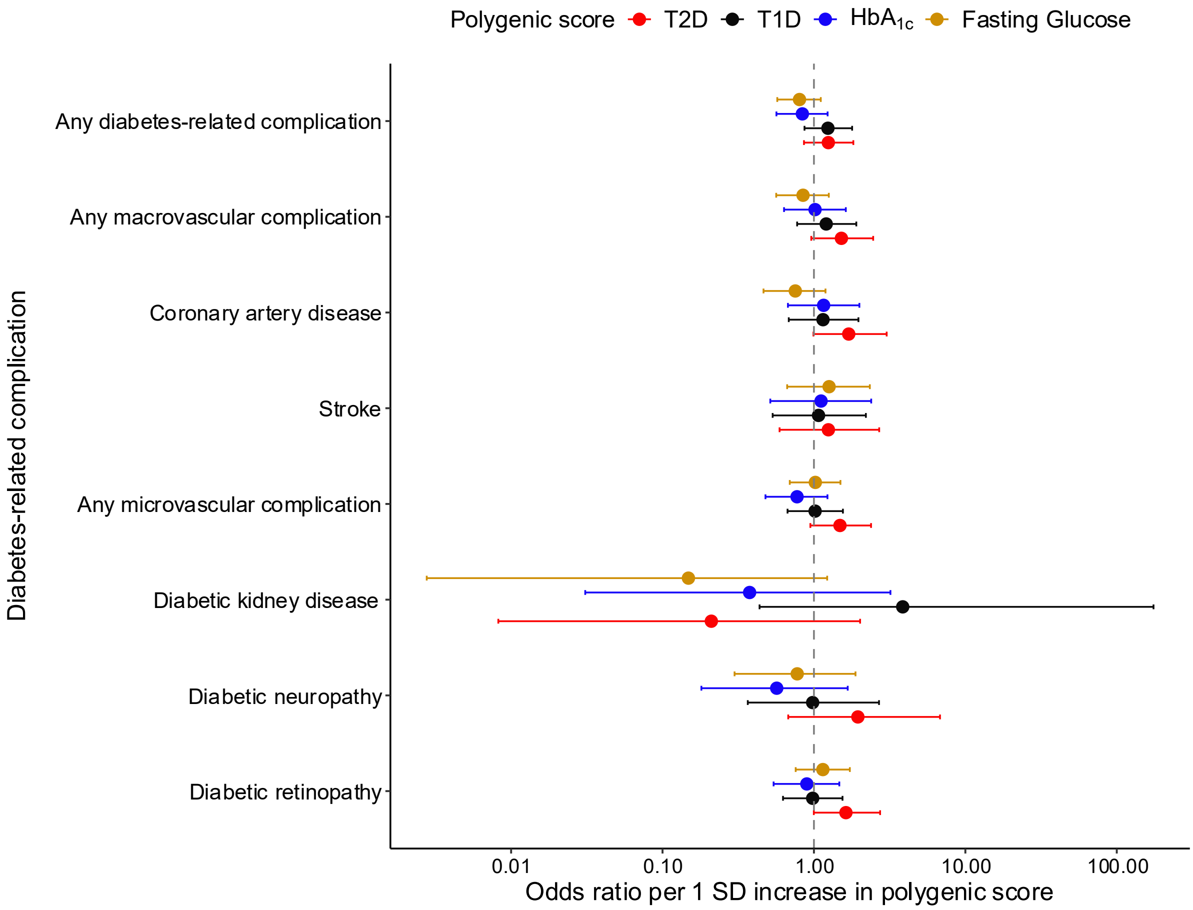

ESM Figure 11: Association of diabetes-related complications with polygenic scores in clinically unselected GCK carriers.

Odds ratios for macrovascular (stroke, coronary artery disease) and microvascular (neuropathy, retinopathy, kidney disease) complications are shown per 1 standard deviation increase in four diabetes-related polygenic scores. Each score was analysed individually in 158 GCK carriers using logistic regression. Dots represent effect estimates, and error bars indicate 95% confidence intervals. Models were adjusted for sex, age, and the first ten ancestry principal components
